# miR-aculous new avenues for cancer immunotherapy

**DOI:** 10.3389/fimmu.2022.929677

**Published:** 2022-09-28

**Authors:** William W. Tang, Kaylyn M. Bauer, Cindy Barba, Huseyin Atakan Ekiz, Ryan M. O’Connell

**Affiliations:** ^1^ Divison of Microbiology and Immunology, Department of Pathology, University of Utah, Salt Lake City, UT, United States; ^2^ Hunstman Cancer Institute, University of Utah, Salt Lake City, UT, United States; ^3^ Department of Molecular Biology and Genetics, Izmir Institute of Technology, İzmir, Turkey

**Keywords:** microRNA, cancer immunity and immunotherapy, exosomes, metabolism, TME (tumor microenvironment), cancer cell, cell death

## Abstract

The rising toll of cancer globally necessitates ingenuity in early detection and therapy. In the last decade, the utilization of immune signatures and immune-based therapies has made significant progress in the clinic; however, clinical standards leave many current and future patients without options. Non-coding RNAs, specifically microRNAs, have been explored in pre-clinical contexts with tremendous success. MicroRNAs play indispensable roles in programming the interactions between immune and cancer cells, many of which are current or potential immunotherapy targets. MicroRNAs mechanistically control a network of target genes that can alter immune and cancer cell biology. These insights provide us with opportunities and tools that may complement and improve immunotherapies. In this review, we discuss immune and cancer cell–derived miRNAs that regulate cancer immunity and examine miRNAs as an integral part of cancer diagnosis, classification, and therapy.

## Introduction

The Global Cancer Observatory predicted a global burden of cancer of 19.3 million new cases in 2020 and projects an annual incidence rate of 28.4 million by 2040. Environmental factors are thought to be the primary contributing factor to the increase in cancer rates and mortality (1–3). Cancer development and progression rely on two key defining features: genetic aberrations within the tumor cells and a dynamic tumor microenvironment (TME) (4). Because mutations in proto-oncogenes and tumor suppressor genes can directly drive of tumorigenesis, therapies targeting these mutations have become a focal point in cancer treatment, starting with imatinib for acute myeloid leukemia in 2001 (5). Tumor cells continuously mutate under the selective pressure of these treatments as an escape mechanism (6). In addition, a complex network of interactions exists between diverse cell types and the non-cellular components comprising the TME (7). As such, tumor escape mechanisms and TME diversity in cancer patient populations lend themselves to further study and therapeutic targets (8–10).

The TME is essential for cancer development and progression, affecting clonal evolution, tumor heterogeneity, migration, invasion, metastasis, vascularization, immune evasion, and therapeutic resistance (11–14). The TME includes tumor cells, endothelial cells, fibroblasts, and immune cells, all of which interact and affect the non-cellular components of the TME (7). Immune cells are dynamic and control or promote tumor progression, as they can constitute a significant portion of the tumor mass (8, 15). Although many immune cells mediate tumor clearance, tumor cells undergo constant evolution under immune selection and eventually escape (16). As tumors undergo immune cell–mediated evolution, they can recruit tumor-promoting immune cells and incapacitate anti-tumorigenic immune cells through metabolic alterations, physical barriers, inhibitory molecules, and extracellular communication (9, 10, 17–19). Our understanding of such pathways has led to therapeutic targeting of tumor-mediated immune suppression and the enhancement of tumoricidal immune cells, leading to the advent of immunotherapy.

Historically, immunotherapy has been a tool to treat unresectable and chemoradiation therapy–resistant cancers. However, it has become the first-line treatment for many cancer types by augmenting the anti-tumor immune responses. Current clinically used immunotherapies utilize and target-specific immune cells, including CD8+ and CD4+ T cells, macrophages, natural killer (NK) cells, and dendritic cells (DCs). Although our understanding of anti-tumor immune responses has progressed substantially, favorable immunotherapy outcomes only apply to a small subset of patients (10, 20–23). A variety of factors differentiate patient subsets in terms of treatment strategies and survival outcomes, including tumor stage, location, mutations, neoantigen load, and gene signatures. Even with defined patient subsets, the current clinical classifications are insufficient, leading to incredibly variable responses to immune-modulating therapies in clinical trials. Thus, there is an ongoing search for new biomarkers and molecular targets for classifying patients and leveraging critical mechanisms for therapeutic benefit. In this context, significant effort has been devoted to studying non-coding RNAs, which can regulate key cellular mechanisms in tumor cells and the immune cells within the TME. Among the non-coding RNA species, microRNAs (miRNAs) have shown great potential as biomarkers and novel therapeutic agents, as they play complex regulatory roles in all cell types within the TME (9, 24–27).

miRNAs are a diverse group of regulatory RNA molecules conserved across multiple taxa with an average length of 22 nucleotides (28–34). Most human miRNAs are found in the intronic regions and transcribed by RNA Pol-II (35). In the canonical miRNA biogenesis pathway, the primary transcript is processed by the microprocessor complex, Drosha/DGCR8, and exported to the cytosol *via* exportin 5 (28). This processed transcript, now termed pre-miRNA, is then edited by DICER to generate the mature miRNA duplex consisting of a 3′ and a 5′ partner (36). On the basis of its thermodynamic properties, one of these strands (the “guide” strand) is loaded onto the Argonaute (AGO) family of proteins, forming the miRNA-induced silencing complex (miRISC), whereas the complementary “passenger” strand is often degraded (36, 37). The rates of miRNA biogenesis and decay in fruit fly cell lines demonstrate that miRNAs are among the fastest produced and longest-lived transcripts (38). The reported median half-life of the AGO-bound guide miRNA strands was 11.4 h, whereas the unbound passenger miRNA strands had a median half-life of ~41 min (38). In mouse fibroblasts, many mature guide miRNAs have half-lives longer than 24 h, whereas passenger miRNAs are quickly turned over with a half-life of 4–14 h (39). Notably, these works reveal heterogeneity in miRNA kinetics, suggesting differential regulation of miRNAs to accommodate cellular needs. Although the primary miRNA transcript levels often positively correlated with their mature counterparts (40), AGO-loading of the guide miRNA strand is a kinetic bottleneck, ensuring faithful miRISC formation (38). Properly formed miRISC subsequently targets mRNAs based on sequence complementarity between the miRNA seed region (nucleotides 2-7) and the 3′–untranslated region (3′-UTR) of mRNA, leading to translational repression and degradation of mRNA (29). A single miRNA can have hundreds of mRNA targets; moreover, 60% of the protein-coding genes in the human genome are thought to be targeted by miRNAs (41). Interestingly, mRNAs can contain multiple miRNA target sequences, and the spatial proximity of these sites can synergistically increase the miRISC-mRNA binding affinity (42). The biochemical basis of this cooperation was recently described and involves TNRC6, an AGO-binding scaffold protein that recruits RNA deadenylation complex (43). Thus, it is conceivable that miRNAs and mRNAs co-evolved to modulate the protein output in a cell. Because multiple genes in a pathway can be simultaneously controlled by miRNA networks, miRNAs can centrally regulate the cellular phenotypes (44). In the context of anti-tumor immunity, these characteristics make miRNAs attractive biomarkers for disease classification and risk assessment and as potential targets to improve clinical outcomes.

Studies have utilized miRNAs to predict immunotherapy responses in humans and as therapeutic agents to augment immune responses or repress tumor cell function in preclinical models (24, 45–47). However, the mounting clinical and preclinical studies highlight our incomplete understanding of miRNAs. Some aspects of miRNAs in cancer immunity have been reviewed (48), but this manuscript provides a comprehensive up-to-date assessment of immune and tumor cell–derived miRNAs that control cancer immunity through intrinsic and extrinsic mechanisms. First, we will examine the role of miRNAs in tumor-infiltrating immune cell fate and function. In addition, we will explore how miRNAs expressed in tumor cells control immune responses and immunological cell death. Finally, we will bridge clinical and preclinical miRNA research through the lens of immunotherapy, discussing the use of miRNAs as biomarkers and future immunotherapy targets by examining current literature on tumor- and immune cell–specific miRNAs.

## Immune cells within the TME

The immune system plays a critical role in cancer progression, as each cancer type has a unique immune cell profile determining patient outcomes (49). Immune cells, such as DCs, macrophages, NK cells, and T cells, work together to eliminate malignant cells. However, cancer cells suppress anti-tumor responses. Tumor-mediated immune suppression co-opts the pathways our immune system relies on for avoiding autoimmunity, and achieving a therapeutic window requires balancing two competing interests: tumor immunity versus autoimmunity. In this particular context, miRNAs may be the key to immune-mediated tumor clearance, as miRNAs repress genes subtly (approximately two- to four-fold changes) and preferentially repress dosage-sensitive targets (50–52). In addition, one miRNA can variably target hundreds of genes depending on the immune cell and the biological context (53, 54), allowing miRNAs to fine-tune delicate processes involved in tumor immunity and autoimmunity. This section will describe the features of miRNA-regulated mechanisms in tumor-associated immune cells.

### T cells

T cells, primarily CD8+ cytotoxic T cells, are positively associated with better tumor outcomes, immunity, and immunotherapy responses. The first association between high T-cell infiltration and early-stage cancer was made in colon cancer (55) and gave rise to the concept of immunologically “hot” versus “cold” tumors. A “hot” tumor has high T-cell infiltration, whereas a “cold” tumor has little to no T cells. T-cell infiltration is currently more predictive of patient outcomes than any other clinical metric in colon cancer (56, 57). Specifically, high T-cell infiltration correlated with early-stage colon cancer, favorable immunotherapy response, and tumor control. Conversely, the loss of T cells and the inflammatory response associated with late-stage cancer, poor immunotherapy response, loss of anti-tumor immunity, and poorer patient outcomes (56–58).

**Figure 1:**
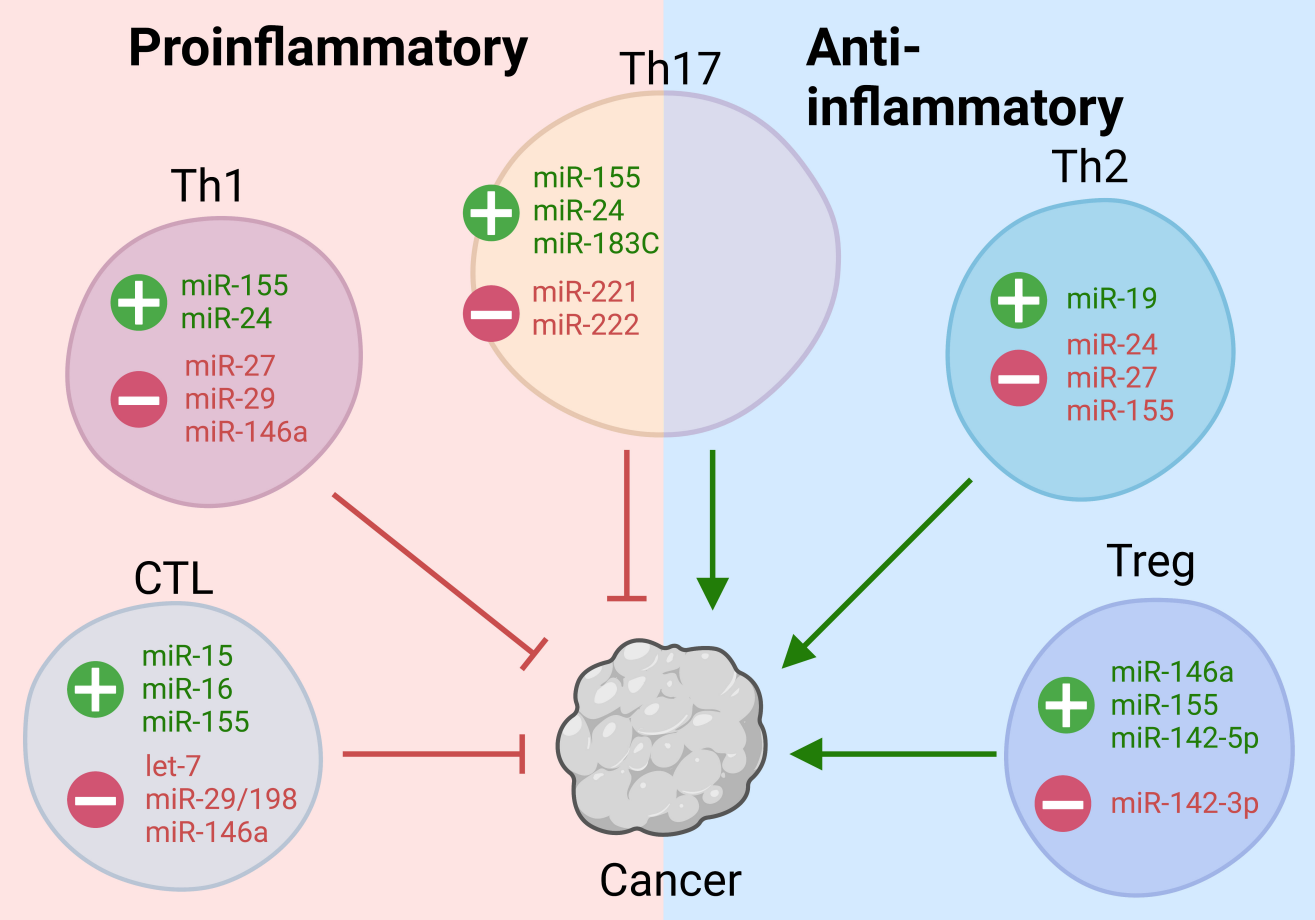
T-cell miRNAs regulate the function of CTLs and helper T-cell subsets in the TME. T-cell miRNAs regulate proinflammatory anti-tumorigenic and anti-inflammatory protumorigenic T cells in the TME. Several miRNAs regulate the fate and function of multiple T-cell subtypes, all of which play defined roles in cancer progression and elimination. These miRNAs and respective targets give mechanistic insights into potential therapeutic targets and agents.

For optimal T-cell–mediated anti-tumor immunity, T cells must recognize an antigen presented on major histocompatibility complexes (MHC) through the T-cell receptor (TCR) and receive a co-stimulatory signal through CD28 engagement with CD80/CD86. However, over-stimulation of T cells with αCD28 monoclonal antibody (mAb) led to severe lymphotoxicity and cytokine storm during a phase I clinical trial (59). miR-214 is upregulated in T cells upon engagement of CD28 and promotes Akt-mediated proliferation following activation. Unlike αCD28-mediated cytokine storm in humans, mice reconstituted with bone marrow cells constitutively expressing miR-214 displayed augmented T-cell function without cytokine storm (60). Other costimulatory molecules, including 4-1BB, OX-40, GITR, ICOS, and CD40L, are also upregulated upon T-cell activation. Regulation and function of these receptors can involve miRNA networks. For instance, 4-1BB engagement leads to methylation of the miR-21 gene, a known repressor of T-cell activation and cytokine production (61). Targeting these co-stimulatory molecules has clinical promise, and miRNAs have great potential for enhancing T-cell function without the unwanted adverse effects, because they can calibrate the immunological output (**Figure 1** and **Table 1**).

**Table 1:** T-cell miRNAs, targets, and functions.

**T-cell activation**			
***miRNA***	***Target***	***Function***	***Reference***
miR-214	Pten	Promotes Akt-mediated proliferation with CD28 engagement	(60)
miR-21	N/A	4-1BB engagement leads to methylation of miR-21 host gene, a repressor of T-cell activation and cytokine production	(61)
**Cytotoxic T lymphocytes (CTLs)**			
***miRNA***	***Target***	***Function***	***Reference***
miR-15a/16	mTOR	Promotes CTL infiltration into tumors, IFN-γ production, CD8+ T-cell activation, and overall survival in a murine glioma model	(62)
let-7	Myc/Eomes	Inhibits CD8+ T-cell proliferation, effector CTL activation, and production of IFN-γ, PFN, and GZMB	(63)
miR-155	Socs1 Ship-1 N/A	Enhances CTL-mediated anti-tumor immune output	(47) (64) (45, 46)
miR-29	Tbet/Eomes	Inhibits CTL function	(65–67)
miR-29/miR-198	JAK3, MCL-1	Promotes apoptosis of CTLs and immune dysfunction	(68)
**Th1 cells**			
***miRNA***	***Target***	***Function***	***Reference***
	N/A		(45, 46)
miR-155	Ship-1	Promotes IFN-γ production from Th1 cells and anti-tumor immunity	(64)
	IFNyR-alpha		(69)
miR-29	Tbet		(65)
miR-27	N/A	Suppresses IFN-γ production from Th1s	(70)
miR-146a	N/A		(64)
miR-24	TCF1	Promotes IFN-γ production from Th1s	(71, 72)
**Th2 cells**			
***miRNA***	***Target***	***Function***	***Reference***
miR-24	IL-4	Inhibits IL-4 production and Th2 polarization	(70, 73)
miR-27	GATA3		
miR-155	c-Maf	Inhibits Th2 function and polarization	(69)
miR-19	PTEN, SOCS-1, A20	Promotes Th2 cytokine production by amplifying PI(3)K, JAK-STAT, and NF-κB signaling	(74)
**Treg cells**			
***miRNA***	***Target***	***Function***	***Reference***
miR-146a	Stat1	Promotes Treg-mediated immune suppression and inhibits Th1 IFN-γ mediated autoimmunity	(75)
	Socs1	Promotes proliferation and homeostatic Treg persistence	(76)
>miR-155	AC9	Downregulated by Foxp3 and inhibits Treg accumulation of cAMP	(77)
miR-142-3p	Tet2	Destabilizes Foxp3 and inhibits Treg persistence	(78)
miR-142-5p	Pde3b	Promotes Treg accumulation of cAMP	(79)
**Th17 cells**			
***miRNA***	***Target***	***Function***	***Reference***
miR-221, miR-222	c-Maf, IL-23R	Promotes an appropriate inflammatory Th17 phenotype with IL-23 stimulation	(80)
miR-155	Ets1	Stimulates expression of IL-23R, IL-23 sensitivity, and Th17-mediated inflammation	(81, 82)
miR-24	TCF1	Stimulates IL-17 production from Th17 cells	(71, 83)
miR-183C	Foxo1	Promotes expression of IL-1R1 and IL-23R and the proinflammatory Th17 cell	(84)
**T-cell exhaustion**			
***miRNA***	***Target***	***Function***	***Reference***
miR-138	PD-1, CTLA-4, Foxp3	Inhibits exhaustion and Treg-mediated immune suppression	(85)
miR-146a	c-Fos (indirect)	Promotes upregulation of PD-1, CTLA-4, Tim-3, and LAG3 and downregulation of anti-tumor function in human T cells	(86)
miR-28	PD-1	Inhibits exhaustion by repressing PD-1 and potentially Tim-3 and BTLA	(87)
	N/A	Regulates overlapping pathways with ICB	(46)
miR-155	PD-L1		(54)
	CTLA-4	Inhibits CD4+ T-cell exhaustion and promotes inflammation	(88)

“N/A” is not available.

#### CTLs and Th1 cells promote anti-tumor immunity

T cells that recognize self-antigens are depleted in the thymus to avoid autoimmunity. However, CD8+ T cells can recognize mutated self-antigens presented on MHC class I (MHCI) of cancer cells. These neo-antigens can prime a strong T-cell response, but immune selection over time skews the tumor cell population to become less immunogenic and inflammatory. Of all T-cell subsets, CTLs are the primary tumoricidal actors, mediating tumor killing *via* perforin (PFN) and granzyme B (GZMB). *In vitro* expanded tumor-infiltrating CTLs have been successfully transplanted back into patients with melanoma, eliciting complete tumor regression in 22% of the patients (89) in autologous cell therapy (ACT) trials. Lymphodepletion with chemotherapy alone or with radiation prior to ACT involving high doses of interleukin-2 (IL-2) enhanced clinical responses to 49% and 52%–72%, respectively. However, immune-related adverse events (irAEs) and treatment-related toxicities, including cytopenia and death, were observed (90–92). In a preclinical model of melanoma, miR-155 overexpression in tumor-specific T cells ablated the need for lymphodepletion and may diminish the associated IL-2-associated iRAEs and treatment-related toxicities (93). Thus, miRNA regulation of T-cell biology may provide a way to fine-tune T-cell responses for optimal anti-tumor immunity without irAEs.

Because the discovery of adaptive immune responses against cancer and IFN-γ–mediated tumor rejection (8), the field has overwhelmingly characterized CD8+ cytotoxic T lymphocytes (CTLs) and CD4+ Th1 cells, both of which produce IFN-γ, as T-cell subsets that mediate anti-tumor immunity (94). IFN-γ and the gene signatures activated by IFN-γ signaling are associated with the best patient outcomes, anti-tumor immunity, and immunotherapy responses (8, 23, 95) and are regulated by miRNAs. In murine glioma, miR-15a/16 knockout (KO) animals have increased CTL infiltration into tumors, IFN-γ production, CD8+ T-cell activation, and overall survival. mTOR degradation mediated by miR-15a/16 was the causative mechanism, and mTOR inhibition with Rapamycin abrogated the enhancement of CTL activity in the absence of miR-15a/16 (62). Let-7 similarly restricted CTL function. Upon T-cell activation, let-7 was induced as a negative feedback regulator and inhibited CD8+ T-cell proliferation, effector CTL activation, and production of IFN-γ, PFN, and GZMB. Constitutive expression of let-7 in CD8+ T cells decreased tumor immunity by repressing Myc and Eomes, two transcription factors (TFs) that promote effector T-cell metabolism and function (63). In the context of CD4+ T cells, miR-29 and miR-27 inhibited Th1 cell function and IFN-γ production (65, 70–72, 96). miR-29 specifically repressed Tbet and Eomes, both of which promote CTL and Th1 function (65–67). CD8+ T cells from renal cell carcinoma overexpressing miR-29 and miR-198 repressed JAK3 and MCL-1, inducing immune dysfunction compared with healthy controls. In addition, inhibition of miR-29 and miR-198 significantly restored JAK3 and MCL-1 expression and prevented apoptosis of CD8+ T cells (68). Lastly, T-cell-specific deletion of anti-inflammatory miRNAs, such as miR-146a, enhanced IFN-γ production from CTLs and Th1 cells, promoting tumor control and anti-tumor immunity (64). Although many miRNAs inhibit T-cell–mediated antitumor immunity, miRNAs can similarly enhance anti-tumor T-cell responses.

miR-155 is among the best and most characterized promoters of anti-tumor immunity in T cells. miR-155 enhances CTL function by promoting proliferation, effector function and memory formation, cytotoxicity against tumor cells, and IFN-γ production. In CD8+ T cells, miR-155 repressed suppressor cytokine signaling-1 (SOCS1), and miR-155 overexpression or SOCS1 inhibition in CTLs further enhanced anti-tumor immunity (47). miR-155 also promoted IFN-γ production from Th1 cells, shaping the entire TME into a proinflammatory and tumoricidal state (45, 46, 64, 69). In CD4+ T cells, miR-24 similarly promoted Th1 polarization and IFN-γ production by repressing TCF-1 (71, 72). Intriguingly, CD4+ T cells lose the ability to become Th2 cells in the absence of Dicer and preferentially skew toward Th1 cells and IFN-γ production, suggesting that miRNA networks are poised to inhibit a Th1-like state (97). There is promising therapeutic potential in altering miRNA expression, as overexpressing miR-155 promotes T-cell anti-tumor activity against low-affinity tumor antigens (98). However, IFN-γ signaling in tumor cells leads to the upregulation of programmed death ligand-1 (PD-L1) to inactivate T cells through programmed cell death protein-1 (PD-1) and to enhance protumorigenic T cells. Thus, miRNA-targeting therapeutics can be combined with immune checkpoint blockade (ICB) inhibitors to yield the optimal anti-tumor effect.

#### Th2 and Tregs promote immune suppression and tumor progression

In cancer, Th2 and T regulatory (Treg) cells are typically considered immune suppressive. Th2 cells secrete IL-4, IL-10, and IL-13, which promote anti-inflammatory M2 macrophages and inhibit proinflammatory M1 macrophage polarization (99). These Th2 cytokine-polarized M2 macrophages can then recruit additional Th2 and Treg cells, both of which enhance immune suppression through IL-10 and transforming growth factor-beta (TGF-β) secretion, leading to the exclusion of CTLs from the TME (100–103). The IL-4 produced from Th2 cells can also induce GATA3, a known Th2 TF, reinforcing the Th2 identity (104). In Th2 cells, miR-24 and miR-27 repressed IL-4 and GATA3, respectively, inhibiting Th2 function (70, 73). miR-155, a Th1-favoring miRNA, also inhibited the Th2 state by repressing c-Maf, an IL-4 promoting TF (69). Lastly, miR-19 promoted Th2 expansion and function by repressing PTEN, SOCS-1, and A20 (74). Whereas Th2 cells can enhance autoimmunity and tumor immunity in some instances, Tregs are ubiquitously considered immune suppressive in cancer.

Tregs are an immunosuppressive helper T-cell subset expressing the TF, FoxP3. Tregs primarily secrete immune suppressive molecules such as IL-10 and TGF-β. Furthermore, Tregs sequester IL-2, a cytokine necessary for effector function and expansion of anti-tumorigenic T cells (105) and promote GZMB-mediated CD8+ T and NK cell death (106). Tregs also express very high levels of CTLA-4, which sequesters co-stimulatory signals and inhibits CTL and Th1 responses. Instead of inducing anergy as in CD8+ CTLs, PD-1/PD-L1 engagement uniquely enhances the expansion of Tregs and creates a positive feedback loop for Treg differentiation. In addition, Treg proliferation and homeostatic persistence are mediated by miR-155 repression of Socs1 (76). These factors all contribute toward Treg-mediated immunosuppression (106, 107), and experimental depletion of Tregs promotes tumor regression and enhances IFN-γ production by CTL and Th1 (108–110). Immunosuppressive miRNAs, such as miR-146, promote Treg suppression through Stat1 repression, and loss of miR-146a in Tregs led to increased Th1 IFN-γ production (75). Tregs also induce immune suppression by transferring intracellular cAMP into other conventional T-cell types through the formation of contact-dependent gap junctions (111). Increased Foxp3 expression leads to higher accumulation of cyclic adenosine monophosphate (cAMP) by downregulating miR-142-3p, which is a negative regulator of adenylyl cyclase 9 (AC9), a cAMP-generating enzyme (77). miR-142-3p also destabilizes Foxp3 by repressing Tet2, and deletion of miR-142-3p restores Tet2 expression and Treg persistence, resulting in less severe autoimmune disease (78). However, miR-142 deletion in Tregs led to severe lethal multiorgan autoimmunity, highlighting miR-142’s critical role in controlling the immune response. This autoimmune phenotype was attributed to the loss of the miR-142-5p, the complementary mature isoform from the miRNA hairpin structure. Rather than mediating the loss of intracellular cAMP, miR-142-5p promoted cAMP accumulation in Tregs by repressing Pde3b, a cAMP-degrading phosphodiesterase (79). Much like Tregs, tumor cells similarly transfer cAMP into effector T cells through gap junctions to suppress the anti-tumor function (112). Although CTL and Th1 enhancement is a pillar of immunotherapy, targeting the immunosuppressive T cells is of equal importance, as there are instances where immunotherapy induce Treg proliferation, causing hyper progression of cancer (113).

#### The divergent role of Th17 cells in tumor immunity

Th17 cells are proinflammatory T cells that induce autoimmunity and responses against fungi. However, their role in anti-tumor immunity remains controversial. In a preclinical model using enterotoxigenic *Bacteriodes fragilis* (ETBF), Th17 cells were required for tumor formation (114); whereas, others have demonstrated the involvement of Th17 cells in promoting anti-tumor immunity (115). The divergent role of Th17 cells may be dependent on the cytokine milieu. Th17 cells induced by TGF-β and IL-6 can prevent autoimmunity and produce IL-10 (116). Induction with IL-1β, IL-6, and IL-23 is associated with chronic inflammation, enhanced autoimmunity, and anti-tumor immunity (115, 117). These Th17 cells not only secrete IL-17, but they also acquire Th1-like features, such as IFN-γ production, which helped promote the anti-tumor response (115, 118). Th17 cells also induce a CTL response by expanding CD8a+ DCs, a cell type critical for cross-presenting tumor antigens to CD8+ T cells (118). TGF-β and IL-23 signaling axes promote c-Maf and Tbet expression in Th17 cells, respectively. TGF-β–induced Th17 cells produce significantly more IL-10 and behave as immunosuppressive cells, whereas IL-23–induced Th17 cells have greater autoimmune potential (119). miR-221/222 is among the key regulators of this contrasting Th17 phenotype. Upon IL-23 stimulation, Th17 cells upregulate miR-221/222, resulting in repressed c-Maf and IL-23R to maintain a balanced Th17 inflammatory response. In the absence of miR-221/222, IL-23 stimulation led to c-Maf and IL-23R upregulation, which promoted a feed-forward loop that strengthened the proinflammatory Th17 response. Conversely, TGF-β signaling inhibited miR-221/222 expression, derepressing c-Maf and promoting Th17 homeostasis and persistence without autoimmune disease (80). Although miR-221/222 modulates the dichotomous Th17 phenotypes through c-Maf, miR-155, miR-24, and the miR-183-96-192 cluster (miR-183C) primarily promote inflammatory Th17 functions. miR-155 represses Ets1, a negative regulator of IL-23 receptor (IL-23R), promotes IL-23 sensitivity, and enhances Th17-mediated inflammation (81, 82). miR-24 inhibits TCF-1, another negative regulator of Th17 cells, and promotes IL-17 production, Th17 differentiation, and Th17-mediated autoimmunity (71, 83). Lastly, IL-23–induced Th17s upregulate miR-183C, which promotes inflammatory Th17 cells by repressing Foxo1, a negative regulator of IL-1R1 and IL-23R. miR-183C expression is correspondingly inhibited in Th17s upon TGF-β signaling (84). Collectively, IL-23 promotes while TGF-β inhibits autoimmunity, and these effector molecules induce distinct miRNAs expression patterns. Thus, miRNA signatures may distinguish distinct tumor-associated Th17 subsets and may explain the controversial role of Th17s in tumor immunity.

#### Chronic activation leads to exhaustion and tumor inactivation of T cells

**Figure 2:**
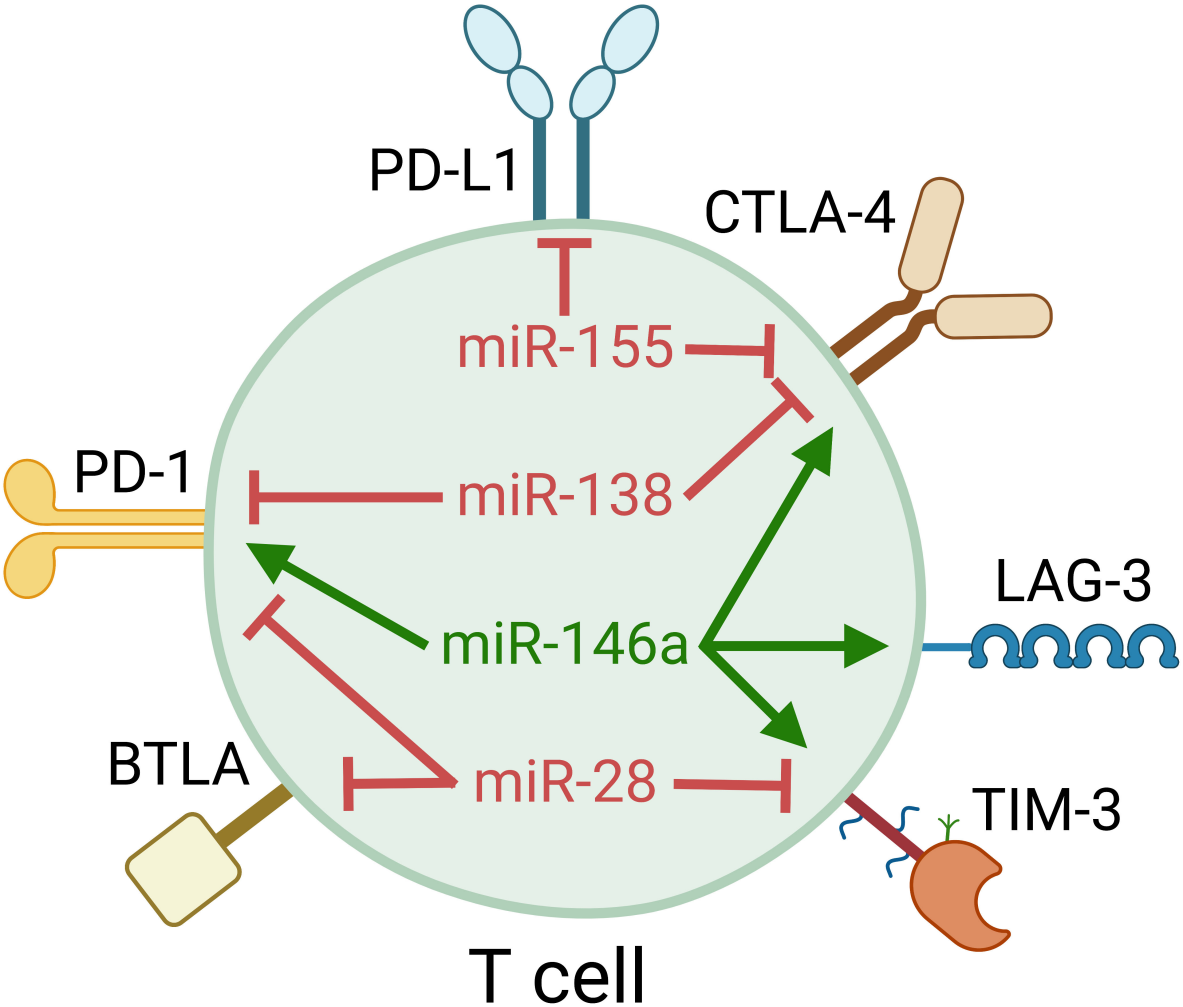
miRNAs regulate T-cell inhibitory checkpoint molecules many of which are targets of clinical immune checkpoint blockade. Each miRNA can target multiple checkpoint molecules, providing a novel tool to target multiple nonoverlapping pathways.

Long-term activation leads to T-cell exhaustion or anergy, which is a mechanism of inactivating T-cell responses. Checkpoint molecules, such as PD-1 and CTLA-4, induce T-cell anergy upon engagement with their respective ligands, preventing autoimmunity and anti-tumor immunity (120, 121). PD-1 is among the key immunomodulatory molecules expressed on the surface of T cells after activation. A state of anergy or exhaustion is induced upon interaction with PD-L1 (B7-H1) and PD-L2 (B7-DC) ligands, which are expressed on myeloid cells, tumor cells, stromal cells, and even other T cells (122–125). CTLA-4, on the other hand, is upregulated later during T-cell activation in lymphoid tissue and binds, trans-endocytoses, and degrades CD80/CD86 to prevent CD28 co-stimulation (126). The loss of co-stimulatory signaling results in T-cell immune tolerance and loss of tumor immunity. As such, blocking these immune checkpoint molecules using mABs led to the most successful and widely used class of immunotherapy, ICB therapy, which is used to treat many cancer types (127, 128). However, a significant portion of the patients treated with α-PD-1 therapy experience irAEs, necessitating the optimization of a proper therapeutic window for better outcomes (129). Because the initial discovery of checkpoint molecules, CTLA-4 and PD-1, there are now several other emerging immune checkpoint molecules that play similar roles, including lymphocyte-activation gene-3 (LAG-3), T-cell immunoglobulin and mucin-domain containing-3 (TIM-3), T-lymphocyte attenuator (BTLA), and V-domain immunoglobulin suppressor of T cell activation (VISTA). Studies now suggest that miRNAs regulate some of these pathways and can be potential therapeutic targets. In human CD4+ T cells, miR-138 represses PD-1, CTLA-4, and FoxP3 expression, and *in vivo* administration of miR-138 mimics to tumor-bearing mice led to enhanced T-cell–mediated tumor immunity and survival (85). Inhibition of miR-146a in exhausted human T cells also led to increased production of IFN-γ and T-cell cytotoxic molecules, including GZMB and PFN, and to downregulation of PD-1, CTLA-4, TIM-3, and LAG-3 (86). This correlates with miR-146a diminishing T-cell–mediated immunity in murine melanoma (64). In addition, miR-28 represses PD-1 and can potentially degrade Tim-3 and BTLA, as inhibition of miR-28 resulted in PD-1, Tim-3, and BTLA upregulation (87). Lastly, miR-155 repressed CTLA-4 and PD-L1 in CD4+ T cells (54, 88). In addition to directly regulating checkpoint molecules, miR-155 also promotes T-cell–mediated tumor immunity through overlapping pathways with ICB (46). Taken together, miRNAs simultaneously impact multiple immune checkpoints and may be pursued as novel therapeutic agents or targets (**Figure 2**).

### NK cells

During T-cell–mediated immune selection, cancer cells are under pressure to present low levels of tumor antigens on MHCI to escape T-cell–mediated cytotoxicity. As a second line of defense, NK cells recognize the cancer cells that lose MHCI expression (130, 131). Unlike CTLs, self-MHCI induces tolerance and inhibits NK-cell–mediated cytotoxicity by binding surface inhibitory receptors such as inhibitory killer gene Ig-like receptors (KIRs) in humans and Ly49 in mice (132). This “missing-self” mechanism of recognition allows NK cell to distinguish healthy cells from cancerous ones (132–134). A network of miRNAs regulates NK cell development, and some miRNAs, including miR-146a-5p, were shown to control NK cell activation by inhibiting KIRs, KIR2DL1 and KIR2DL2 (135).

In addition to inhibitory receptors, NK cells also express stimulatory receptors such as natural killer group 2D (NKG2D) that bind to tumor antigens, RAE1 and H60, to mediate tumor rejection (136). In fact, loss of NKG2D led to increased tumor development (137). In humans, specific NKG2D haplotypes demonstrate the relevance of surface stimulatory receptors in cancer outcomes. For instance, the high-activity–related HNK1 haplotype is associated with enhanced NK cell cytotoxicity; whereas, the low-activity–related LNK1 haplotype correlated with low cytotoxicity. Notably, the HNK1 haplotype has higher expression of NKG2D, and the LNK1 haplotype has lower expression of NKG2D. The differential NKG2D expression was attributed to the 3′-UTR seed region in LNK haplotype binding to miR-1245 more efficiently and silencing NKG2D at a higher magnitude. Phenotypically, the LNK haplotype resulted in decreased cytotoxicity against HPV-infected cervical cancer cells and higher susceptibility to TGF-β–mediated NKG2D suppression (138). When NK cells receive anti-inflammatory TGF-β signaling, miR-1245 functions to repress NKG2D expression and NKG2D-mediated cytotoxicity. Downregulation of miR-1245 restored NK cell cytotoxicity (139), suggesting NK-cell–specific miRNAs can be targeted to combat cancer cells. TGF-β also inhibits NK cell function by repressing DNAX-activating protein 12 (DAP12), a signal adaptor protein for all activating allelic variants of KIR (aKIR). Mechanistically, TGF-β mediates the degradation of DAP12 by upregulating miR-183 (140). Beyond activating and inhibitory receptor signaling, miRNAs directly influence the production of effector molecules to regulate the NK cell output.

**Figure 3:**
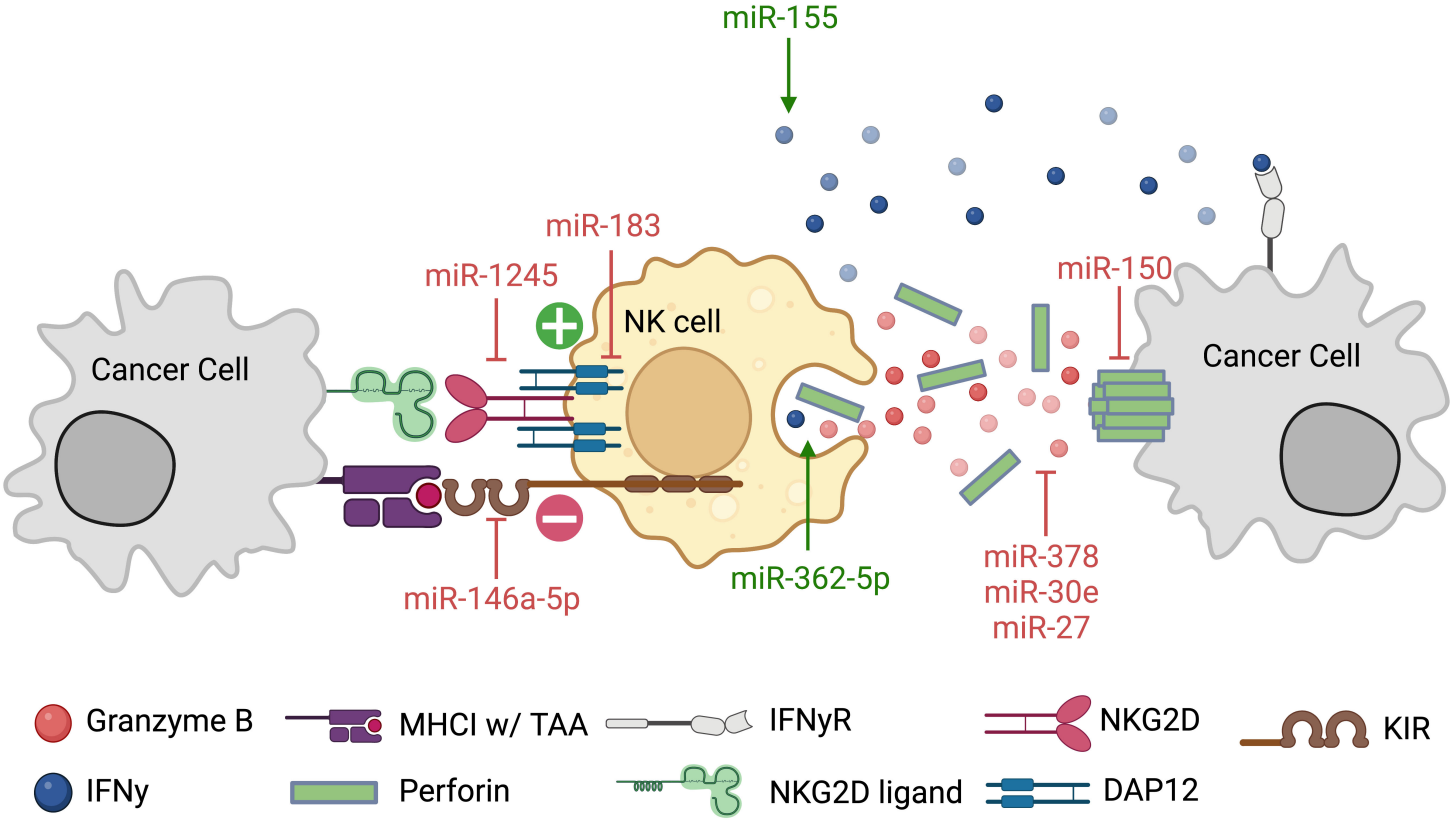
Natural killer cell miRNAs regulate tumor cell recognition and NK cell function. NK cells have many surface stimulatory and inhibitory molecules required for recognizing and targeting cancer cells. Not only do miRNAs regulate these molecules, but they also regulate the adaptor proteins, DAP12, and effector molecules, including IFN-γ, Gzmb, and Pfn.

IFN-γ produced from NK cells plays a unique role in promoting further NK cell accumulation, activation, and cytotoxicity, all of which prevent metastasis in multiple murine tumor models (135). Among the miRNAs that regulate NK phenotypes, miR-155 strongly induces IFN-γ production following IL-12–, IL-18–, or CD16-mediated activation through a mechanism involving SHIP-1 repression (141). Comparably, miR-362-5p represses CYLD, a negative regulator of a nuclear factor kappa-light-chain-enhancer of activated B cells (NF-κB)–mediated inflammatory response. Overexpression of miR-362-5p increased production of multiple effector molecules including IFN-γ, PFN, GZMB, and CD107. Silencing CYLD had the same effect as miR-362-5p overexpression and partially restored function in dysfunctional miR-362-5p KO NK cells (142). IFN-α similarly promotes NK cell accumulation, activation, and cytotoxicity, to protect mice from cancer (143). In fact, IFN-α–activated NK cells downregulate miR-378 and miR-30e, both of which repress GZMB and PFN (144). GZMB and PFN can also be repressed by miR-27, and knockdown of miR-27 increases *in vitro* cytotoxicity, leading to decreased tumor growth in a human tumor xenograft model (145). Lastly, miR-150 represses PFN and results in decreased NK cell cytotoxicity, increased tumor growth, and metastatic burden in mouse lungs (146). In conclusion, the anti-tumorigenic potential of NK cell functions can be regulated by multiple miRNAs, and targeting these miRNAs may offer new avenues or complement existing immunotherapy to improve anti-tumor immunity (**Figure 3** and **Table 2**).

**Table 2:** NK cell, DC, macrophage, and MDSC miRNAs, targets, and functions.

Natural killer (NK) cells			
***miRNA***	***Target***	***Function***	***Reference***
miR-146-5p	KIR2DL1, KIR2DL2	May augment NK cell killing of tumor cells	(135)
miR-1245	NKG2D	Decreases NKG2D-mediated cytotoxicity	(138, 139)
miR-183	DAP12	Inhibits the signal adaptor protein for all activating allelic variants of KIR (aKIR)	(140)
miR-155	SHIP-1	Induces IFN-γ production after IL-12, IL-18, or CD16 mediated activation	(141)
miR-362-5p	CYLD	Promotes NF-κB–mediated inflammatory response, increasing production IFN-γ, PFN, GZMB, and CD107	(142)
miR-378, miR-30e	GZMB, PRF1	Inhibits NK cell cytotoxicity	(144)
miR-27	Gzmb, Prf1		(145)
miR-150	PRF1		(146)
**Dendritic cells (DCs)**			
***miRNA***	***Target***	***Function***	***Reference***
miR-155	c-Fos	Promotes increased expression of CD86 and CD40 expression and T-cell activation	(147)
	Arg-2	Promotes T-cell function by inhibiting arginine sequestration	(148)
miR-148a, miR-148b, miR-152 (miR-148 family)	CAMKII	Inhibits IL-6, TNFa, and type I and II IFN secretion, and expression of MHCII	(149)
miR-148a	DNMT1	Promotes expression of SOCS1 and inhibits TLR3/4 stimulation of DCs	(150)
let-7i	SOCS1	Inhibits development CD86− DCs that promote generation of Tregs	(151)
miR-30b	Notch1	Stimulated by TGFB and Smad3 pathways and promotes IL-10 secretion and immune suppression	(152)
miR-24, miR-30b, miR-142-3p	N/A	Promotes PDL-1-mediated immune suppression and inhibits antigen uptake and presentation to CD4+ T cells, decreasing IFN-γ production from Th1 cells	(153)
miR-301a	N/A	Decreases IL-12 expression, IFN-γ production by CTLs, and Th1 polarization of CD4+ T cells	(154)
miR-9	Pcgf6	Promotes cDC1 function, CD8+ T-cell priming, expansion of tumor specific CD8+ T cells, and tumor clearance	(155, 156)
**Macrophages**			
***miRNA***	***Target***	***Function***	***Reference***
miR-155	Tspan14, MafB, Inpp5d, Ptprj	Represses transcriptional network that promotes M1 polarization and inhibits M2 polarization to promote tumor killing	(157)
	IL-13a1	Prevents STAT-6 phosphorylation and expression of CD23, CCL18, and SOCS1, inhibiting M2 macrophage polarization	(158)
miR-720	GATA3	Suppresses M2 macrophage polarization and function (CCL17, IL-10, and Arg-1)	(159)
miR-125b	IRF4	Increases CD80, CD86, CD40, and MHCII expression in macrophages and IFNyR in CD8+ T cells and promotes anti-tumor immunity	(160)
miR-378-3p	Akt-1	Restricts M2 macrophage proliferation	(161)
miR-511-3p	N/A	Induced upon M2 polarization and inhibits M2-mediated angiogenesis, protumorigenic factors, and tumor growth	(162)
let-7c	C/EBP-delta	Promotes M2 polarization and inhibits M1 gene expression and function, including CCR7, IL-12, iNOS, and MHCII expression	(163)
miR-21	STAT3	Inhibits M1 polarization to promote M2 polarization	(164)
miR-21, miR-29a	N/A	Induced by Ets and promotes M2 polarization, angiogenesis, metastasis, and poorer patient outcomes	(165)
miR-223	Nfat, Rasa1	Induced by Ets and PPARy and promotes M2 polarization, cancer cell invasiveness, and metastasis	(166)
	C/EBP-β	Inhibits M1 polarization	(167)
**Myeloid-derived suppressor cells (MDSCs)**			
***miRNA***	***Target***	***Function***	***Reference***
miR-210	IL-16, CXCL12	Induced by HIF-1α and inhibits T-cell function and IFN-γ production by promoting Arg-1 and inhibiting IL-16/CXCL12 expression	(168)
miR-155	HIF-1α	Inhibits MDSC-mediated immune suppression/tumor growth *via* Arg-1, iNOS, VEGF, MMP2, and MMP9 and MDSC accumulation in tumors	(169)
miR-155	SOCS1	Promotes MDSC proliferation/function, increases *Arg-1*, *Mmp-9*, *Vegf*, and *iNOS* expression, and enhances T-cell immune suppression	(170)
miR-155, miR-21	SHIP-1, PTEN	Promotes STAT3 phosphorylation, a critical TF for MDSC development	(171)
miR-21a	Wdr5	Promotes PMN-MDSC proliferation, expression of Arg-1 and iNOS, and suppression of CD4+/CD8+ T cells and M1 macrophages	(172)
miR-21b	Ash2l		
miR-181b	Mll1		
miR-30a	SOCS3	Increases ROS, ARG-1, and IL-10, inhibiting the proliferation of and IFN-γ production from CD4+ T cells and promoting tumor burden. Also promotes MDSC development and function by promoting STAT3 phosphorylation	(173, 174)
miR-494	PTEN	Increases expression of Arg-1, MMP2, MMP13, and MMP14 and activation of Akt, mTOR, and NF-κB, promoting metastasis and proliferation	(175)
miR-9	Runx1	Promotes Arg-1 expression and MDSC function in mouse and human lung cancer	(176)
miR-34a	N-myc	Promotes MDSC persistence and proliferation, induces terminal differentiation to an M1 phenotype, and restricts tumor growth	(177)

“N/A” is not available.

### Dendritic cells

DCs are antigen-presenting cells (APCs) that prime the immune response more efficiently than any other cell type (178, 179). DCs express MHCI and MHC class II (MHCII) required for antigen cross-presentation to CD8+ T cells and classical antigen presentation to CD4+ T cells, respectively. Upon activation through Toll-like receptors (TLRs), DCs upregulate MHC and co-stimulatory molecules, including CD40 and CD80/CD86, all of which interact with T cells to promote a proinflammatory or anti-tumor response. miRNAs, such as miR-155, can regulate both processes simultaneously, as miR-155 promotes increased CD86/CD40 expression on DCs and improved antigen presentation to CD4+ T cells, enhancing T-cell activation. Mechanistically, miR-155 represses c-Fos, an inhibitor of co-stimulatory molecules and antigen-specific T-cell activation (147). miR-155 also promotes DC-antigen-specific T-cell expansion by repressing Arginase-2 (Arg2), an enzyme that sequesters extracellular arginine, which is an essential metabolite for T-cell function (148, 180–182). After activation, CD4+ T helper cells express CD40 ligand (CD40L), which reciprocally enhances CD80/86 expression after binding to CD40 on DCs. Simultaneous signaling from CD40L and IFN-γ are then required for IL-12 production (183), which stimulates IFN-γ production from T and NK cells, inhibiting metastasis (184).

**Figure 4:**
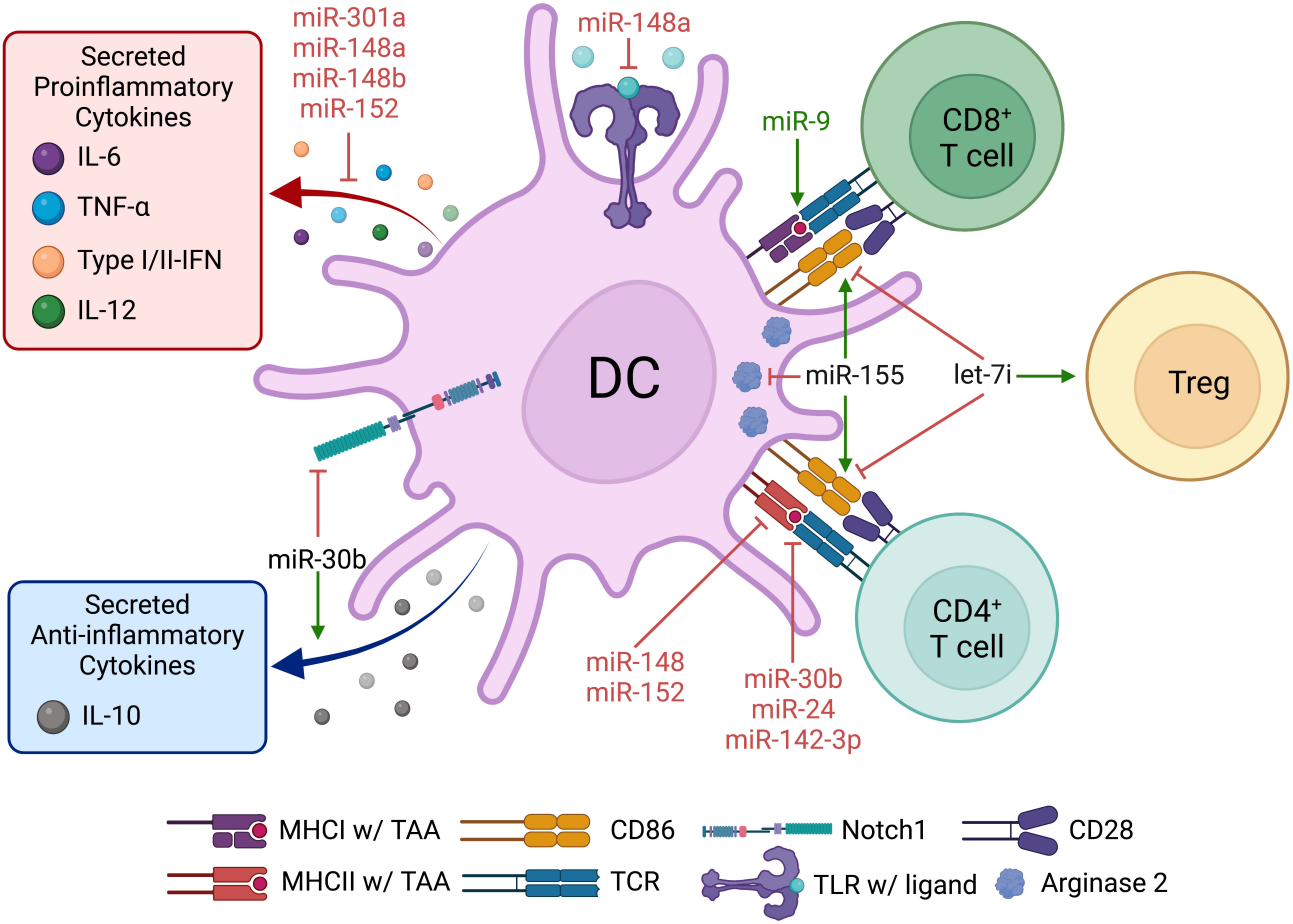
Dendritic cell miRNAs regulate pro- and anti-tumor immune responses in the TME. Dendritic cells are a central hub for priming pro- and anti-tumor immune responses in cancer. Many DC miRNAs change TAA presentation to and co-stimulation of T cells, required for T-cell–mediated anti-tumor immunity. Several other miRNAs suppress the proinflammatory functions and promote the immune suppressive function of DCs.

DCs also promote anti-tumor immunity by secreting proinflammatory cytokines, including IL-6, TNFα, and type I and II interferons (IFNs). These inflammatory DCs require calcium/calmodulin-dependent protein kinase II (CaMKII), which is regulated and degraded by miR-148a, miR-148b, and miR-152, members of the miR-148 family (149). Upon TLR3, TLR4, and TLR9 stimulation, miR-148/152 is elevated and leads to decreased proinflammatory cytokine production. Overexpression of miR148/152 restricts expression of MHCII, antigen presentation to CD4+ T cells, and clonal expansion of antigen-specific T cells. Inhibition of miR-148/152 promotes the DC proinflammatory profile (149). Tumor-associated DCs (TADCs) express elevated miR-148a, which represses DNA methyltransferase (DNMT) 1 and causes subsequent hypomethylation of SOCS1, a suppressor of TLR signaling. Thus, miR-148a–mediated increase in SOCS1 promotes resistance against TLR3 and TLR4 stimulation. Inhibition of miR-148a leads to increased DNMT1 expression and decreased SOCS1, reversing the suppressed phenotype (150). Following these findings, a miR148a inhibitor, poly I:C (TLR3 agonist), and tumor antigen were then packaged together in a DC-based cationic polypeptide micelle nanovaccine, which promotes enhanced anti-tumor immunity and survival by expanding mature DCs and suppressing Treg and myeloid-derived suppressor cell (MDSC) development (150). Similarly, administration of a TLR3 agonist, ARNAX, with a tumor-associated antigen (TAA) enhanced anti-tumor immunity and sensitized ICB-resistant tumors to anti–PD-L1 therapy (185). The preclinical ARNAX and miR-148a nanovaccine models demonstrate the capacity of miRNAs in DCs to optimize anti-tumor immunity and ICB responses (**Figure 4** and **Table 2**).

Cancer-mediated DC activation requires fine-tuning, as it can induce T-cell anergy through PD-L1 expression and induce Tregs (186–188). Within the murine CRC tumors, DCs express the highest levels of PD-L1, which can inhibit T-cell–mediated anti-tumor immunity. Interestingly, DC PD-L1 expression was induced by IFN-γ and CD8+ T cells and required for anti–PD-L1 therapy sensitivity (186). This may be a mechanism of immune evasion, and α-PD-L1 therapy may be a way to overcome the immune suppression. Compared with tumor-free rodents, tumor bearing rodents have expanded immature myeloid DCs (IMDCs) that preferentially prime and expand Tregs through TGF-β signaling (186). miRNA, let-7i, is involved in DC induction of Tregs. In the absence of let-7i, DCs skew into CD86+ and CD86− DC subsets, and the latter subset induces Tregs through the de-repression of SOCS1 (151). TGF-β also differentiates DCs into regulatory DCs, inhibiting T-cell responses. Regulatory DCs rely on miR-30b repression of Notch1 to promote IL-10 secretion and immune suppression (152). miR-30b along with miR-24 and miR-142-3p inhibit antigen uptake and presentation to CD4+ T cells. This defect leads to decreased IFN-γ production from Th1 cells and increased PD-L1–induced T-cell anergy (153). Lung tumors also induce an anti-inflammatory DC miRNA profile to disrupt transport and cross-presentation of tumor antigen to naïve T cells in mediastinal lymph nodes. In particular, DC miR-301a overexpression decreased IL-12 expression, IFN-γ production by CTLs, and Th1 polarization of CD4+ T cells. Furthermore, deletion of Dicer in dendritic cells resulted in better lung cancer immunity, supporting the notion that tumor cells induce inhibitory miRNAs in DCs (154).

Recently, multiple distinct subsets of DCs have been identified within tumors. Tumor-associated CD103+ DCs, defined as type 1 conventional DCs (cDC1s), are the main source for CTLs priming (189). High cDC1 abundance in tumor draining lymph nodes is necessary for maintaining a reservoir of tumor-antigen specific TCF-1+ CD8+ T cells. FLT3 ligand (FLT3L)–and α-CD40–treated mice had expanded cDC1 populations and TCF1+ CD8+ T cells in tumor draining lymph nodes, enhancing tumor immunity (190). miRNAs control the formation of cDC1s, as miR-9 is highly expressed in cDC1s under proinflammatory conditions. miR-9 promotes cDC1 function and naïve CD8+ T-cell priming by repressing the transcriptional repressor polycomb group factor 6 (PCGF6). Further, DC miR-9 overexpression enhanced tumor clearance and the expansion of tumor-specific effector CD8+ T cells (155, 156). Since the initial characterization of cDC1s, single-cell RNA sequencing has provided higher resolution of TADC subsets. Currently, cDC1, cDC2, Mo/cDC2, pDC, and DC3 are the key DC populations, and a recent study showed that DC profiles in non–small cell lung cancer (NSCLC) were largely consistent between patients. Notably, mouse and human TADCs showed highly conserved DC states, indicating the translatability of the preclinical findings (191). Although DC states are conserved across patients and between human and mice, further research is needed for understanding the significance of unique miRNA profiles across the DC subsets.

### Macrophages

Macrophages are phagocytic cells with the capacity to engulf and kill microorganisms and are increasingly appreciated for their complex roles in cancer immunity. In the TME, IFN-γ–induced M1s are considered proinflammatory and anti-tumorigenic, whereas anti-inflammatory protumorigenic M2s are polarized by IL-4, IL-10, or IL-13 (100, 102, 192–194). Thus, patient outcomes shift dramatically when tumor-associated macrophages (TAMs) are stratified on the basis of the M1–M2 axis. In breast and colon cancer studies, high M1 infiltration is associated with better survival, and high M2 infiltration is associated with poorer survival (195–198). The dichotomous M1–M2 classification may be overly simplistic and insufficient, especially in the context of tumor immunology, as it only encompasses the extremes of macrophage activation states. For instance, a recent study found no changes in patient outcomes in patients with high M1 macrophage association and M1/M2 ratios. Despite no changes in survival, a high M1 macrophage association and an M1/M2 ratio resulted in improved IFN-γ response, lymphocyte infiltration signature, and an anti-tumorigenic immune landscape, including increased CD8+ T cells, Th1 cells, NK cells, and DCs (199). Because of our evolving perception of TAMs, understanding macrophage plasticity in tumors is vital in realizing the full therapeutic potential of macrophages.

The M1–M2 axis is controlled post-transcriptionally by miRNAs (200). In fact, miRNAs are critical for macrophage development, persistence, and function. Loss of Dicer in hematopoietic stem cells (HSCs) leads to the depletion of mature myeloid cells and their precursor stem cells (201). miR-155 is a key regulator of inflammation in macrophages and is induced in the presence of TLR3 agonist (poly I:C) or type I and type II IFNs through MyD88/TRIF or by TNF-α autocrine signaling, respectively (202). Both pathways of macrophage induction promote M1s and require JNK-dependent induction of miR-155 (202, 203). Further, miR-155 globally promotes M1 transcriptional networks by repressing *Tspan14*, *MafB*, *Inpp5d*, and *Ptprj* (157). Depletion of miR-155 under M1 polarizing conditions leads to M2 polarization, whereas transient miR-155 overexpression under M2 polarizing conditions leads to M1 polarization and increased tumor killing *in vitro* (204), suggesting that miR-155 is critical for macrophage differentiation. In addition, a polypeptide nanocomplex containing miR-155 repolarized M2 TAMs to the M1 state in a mouse model of melanoma. This nanocomplex increased miR-155 expression in TAMs by 100–400 fold, promoted T and NK cell priming, and induced tumor regression (205). These studies highlight the necessity of miR-155 in M1 polarization and demonstrate the potential of experimental miRNA manipulation, altering macrophage function and plasticity.

Functionally, M1 macrophages are identified by increased expression of TNF-α, inducible nitric oxide synthase (iNOS or NOS2), CD40/80/86, and MHCII expression. Conversely, M2 macrophages are identified by their ability to suppress anti-tumor responses by producing IL-10 and TGFβ, recruiting Tregs and Th2 cells, supporting fibrosis to exclude CTLs, sequestering L-arginine through Arginase-1 (Arg-1), and expressing immune checkpoint molecules (100, 102, 192–194). A component of maintaining the M1 state depends on restricting the development and function of the M2 macrophages. Supporting this view, miR-155 degrades IL-13Ra1 and subsequently inhibits STAT6 phosphorylation and CD23, CCL18, and SOCS1 expression, all critical drivers of M2 state (158). Other miRNAs, including miR-720, miR-125b, miR-378, and miR-511, also suppress M2 macrophages. By degrading GATA3, miR-720 inhibits the expression of CCL17, IL-10, and Arg-1 (159). Further, miR-125b inhibits M2 polarization through the repression of IRF4 and, when overexpressed in macrophages, increases *in vitro* CD8+ T-cell priming, tumor clearance, and CD80/86, CD40, MHCII, and IFN-γ receptor (IFN-γR) expression (160). miR-378-3p restricts M2 macrophage proliferation by repressing Akt-1 (161). Lastly, miR-511-3p overexpression disrupts angiogenesis and M2 protumorigenic functions, inhibiting tumor growth (162). However, miR-125b-5p, miR-378-3p, and miR-511-3p are rapidly upregulated upon IL-4–induced M2 polarization, highlighting the role of miRNAs in counteracting M2 function despite a positive correlation with M2 macrophages, possibly as part of a negative feedback loop.

**Figure 5:**
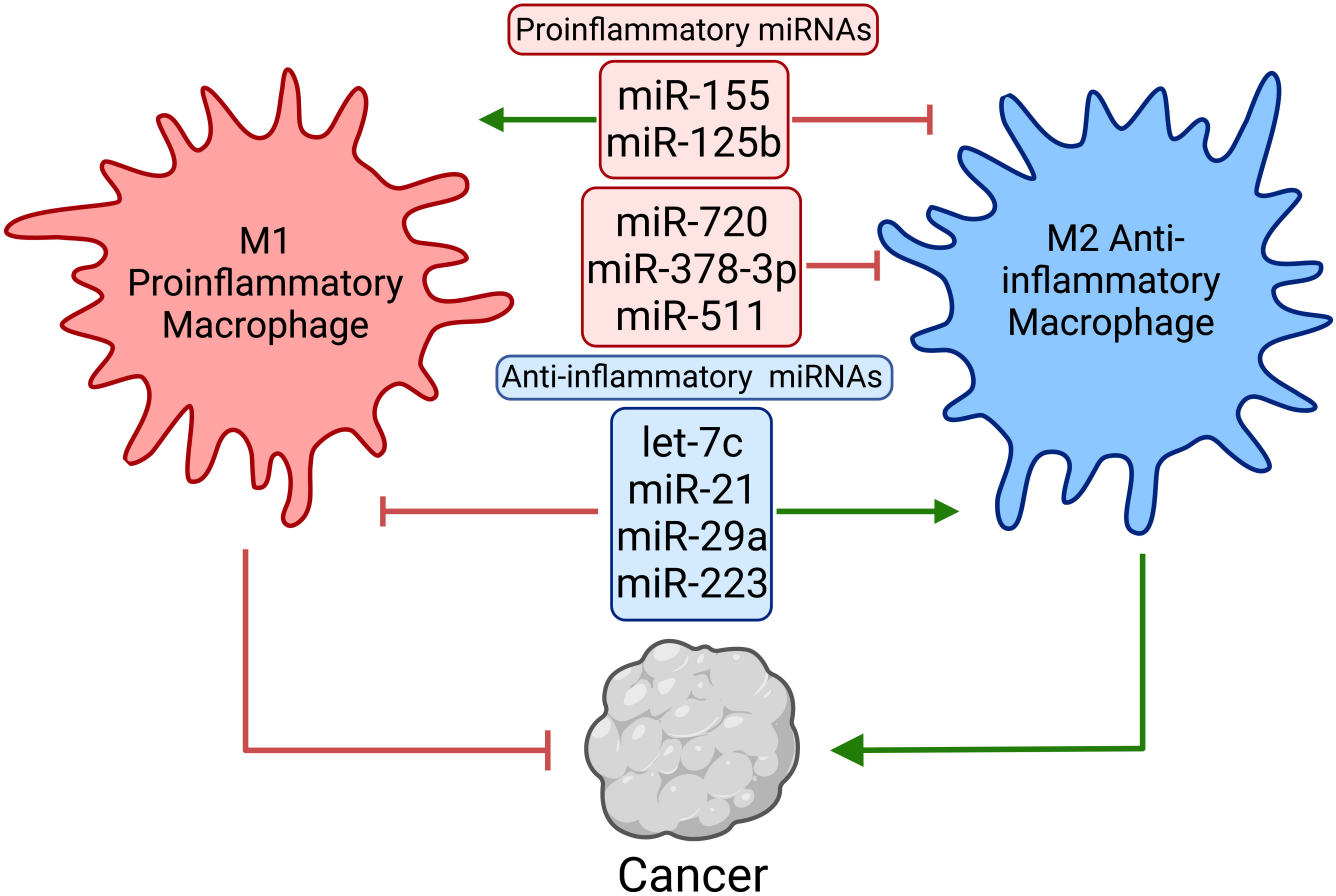
Macrophage miRNAs regulate pro- and anti-inflammatory macrophage skewing and function. Macrophages exist on a continuum on the M1–M2 axis. M1 macrophages are considered proinflammatory and promote anti-tumor functions, whereas M2 macrophages are anti-inflammatory and immune suppressive. Macrophage miRNAs post-transcriptionally reinforce the identity of and regulate the effector function of M1/M2 macrophages.

Like maintaining the M1 state, M2 differentiation also involves reciprocal inhibition of the opposite activation extreme. For instance, let-7c is highly expressed under M2 conditions and promotes M2s by repressing C/EBP-delta (163). Thus, let-7c stabilization during M1 conditions leads to diminished CCR7, IL-12, iNOS, and MHCII expression and increased M2 gene expression. Conversely, the loss of let-7c under M2 conditions led to decreased ARG1, FIZZ1, and YM-1 expression, all of which are characteristic of M2 macrophages (163). In addition to its immune suppressive function, prostaglandin E_2_ (PGE2) induces M2 macrophage polarization. PGE2 and its downstream effector molecules inhibit miR-21. Upon miR-21 depletion, M1 macrophage polarization is impaired, and M2 macrophage polarization is enhanced even in the absence PGE2, suggesting a reliance on the downregulation of miR-21 for M2 polarization. Notably, the loss of miR-21 led to derepressed STAT3, a potent inhibitor of M1 macrophage function (164). The reciprocal inhibition mechanism between M1/M2 states indicates that induction of anti-tumorigenic M1 differentiation should also involve restricting the M2 phenotype. Thus, the critical miRNAs controlling macrophage differentiation and functional pathways, including miR-155 and others, may serve as potential therapeutic targets (**Figure 5** and **Table 2**).

Thus far, we have discussed miRNAs that enhance or restrict the identity of macrophages. However, it is also important to examine how critical TFs alter miRNAs and macrophage function. During cancer progression, C-C motif chemokine ligand 2 (CCL2) and colony-stimulating factor 1 (CSF1) recruit and maintain macrophages in the TME (206), and CSF1-recruited TAMs express TFs, including Ets2, to promote angiogenesis, tumor growth, and breast cancer metastasis (207). miRNAs can similarly drive angiogenesis, tumor growth, and metastasis, as complete ablation of mature miRNAs in mature myeloid cells and macrophages led to decreased metastasis, vascular formation, and proliferation of tumor cells (165). Notably, miR-21, miR-29a, miR-142-3p, and miR-223 were upregulated in TAMs during late mouse metastatic breast cancer and melanoma models and contained ETS binding motifs. Upon ETS deletion, these four miRNAs were concordantly diminished. miR-21 and miR-29a overexpression led to increased angiogenesis and tumor cell proliferation by degrading anti-angiogenic factors and M1-related genes while promoting the expression of M2 genes. miR-21 and miR-29a expression was also higher in myeloid cells in metastatic lesions and correlated to metastatic burden and poorer patient outcomes (165). Although this work did not elucidate the impact of miR-223, several other studies have examined its role in promoting metastasis and M2 macrophage function. In one study, M2 macrophages transferred miR-223 into breast cancer cells through microvesicles, potentiating cancer cell invasiveness and their metastatic potential (208). Under tumor-promoting Th2 conditions, macrophages upregulate PPARy and miR-223. PPARy expression is crucial in M2 polarization, and increased PPARy binds to PPARy regulatory elements (PPRE), which is upstream of the pre-miR-223 coding region. Depletion of miR-223 led to the loss of M2 polarization, as its targets, *Nfat5* and *Rasa1*, are derepressed (166). Lastly, miR-223 restricted proinflammatory M1 macrophage cytokine production by repressing C/EBPβ (167). Taken together, miR-223 exemplifies a macrophage-derived miRNA that inhibits proinflammatory macrophages while promoting tumor macrophage trafficking, metastasis, immune suppression, and invasiveness of cancer cells. Pleiotropic miRNAs such as miR-223 and miR-155 are crucial in regulating many axes of macrophage-dependent tumor biology. Their unprecedented specificity in regulating the macrophage function and plasticity lends miRNAs as promising therapeutic targets or agents.

### Myeloid-derived suppressor cells

**Figure 6:**
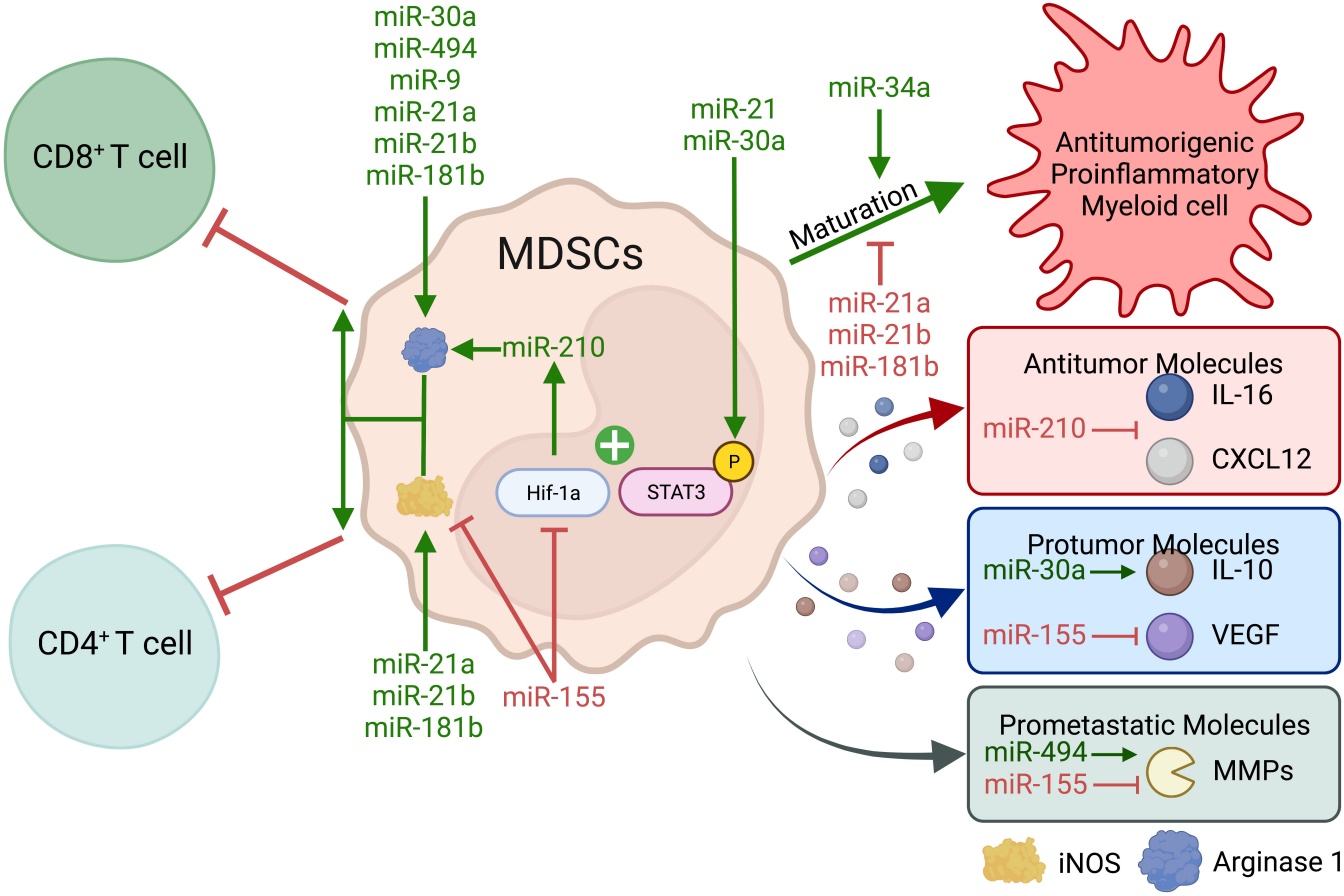
Myeloid-derived suppressor cell miRNAs alter the immune-suppressive capacity of MDSCs. MDSCs have many mechanisms to inhibit anti-tumor T-cell responses and promote tumor progression. Most MDSC miRNAs promote Arginase and iNOS-mediated CD4+ and CD8+ T-cell suppression. Conversely, several miRNAs promote the maturation of MDSCs into proinflammatory myeloid cells with anti-tumor and M1 macrophage functions. miR-155 can inhibit MDSC suppressive function, tumor vascularization, and metastatic potential; although its role in MDSC function is controversial.

Aberrant myelopoiesis during pathology promotes the development of MDSCs. Specifically, prolonged inflammation leads to changes in the bone marrow and spleen that support MDSC development and production (209, 210). Once induced, MDSCs promote an immune-suppressive TME by inhibiting anti-tumorigenic T cells and promoting tumor vascularization *via* vascular endothelial growth factor (VEGF), extracellular matrix modification through matrix metalloproteinases (MMPs), and ultimately tumor progression. Continuous chronic inflammation also leads to the conversion of monocytes and PMNs into MDSCs in peripheral tissue or tumors. High levels of granulocyte-macrophage colony-stimulating factor (GM-CSF), IL-6, and IL-1β stimulate the development of polymorphonuclear MDSCs (PMN-MDSCs), which primarily mediate immune suppression through reactive oxygen species (ROS), Arg-1, and PGE2. M-CSF promotes the development of monocytic MDSCs (M-MDSCs) that mainly utilize nitric oxide (derived from iNOS), IL-10, TGF-β, and PD-L1 to suppress anti-tumor immune responses. Several overlapping developmental factors include TGF-β, VEGF, adenosine, and hypoxia-inducible factor 1 alpha (HIF-1α) (211). However, the field currently faces challenges in distinguishing MDSCs from PMNs and monocytes. Human PMN-MDSCs in peripheral blood from patients with cancer can be identified through lectin-type oxidized LDL receptor 1 (LOX1) (212), but mouse MDSC identification relies on somewhat non-descript surface markers. Therefore, it is difficult to distinguish CD11b+Ly6G+Ly6C− PMN-MDSC from a neutrophil and CD11b+Ly6G-Ly6C+ M-MDSC from a monocyte. Because of their defined roles in different cell types, miRNAs may provide a means to distinguish MDSCs and provide mechanistic insights into MDSC function (**Figure 6** and **Table 2**).

One aspect of MDSC development and function relies on the hypoxic TME, which induces HIF-1α and enhances miR-210 expression in MDSCs. Hypoxia-induced MDSCs and MDSCs with miR-210 overexpression during normoxia similarly inhibited T-cell function and IFN-γ production by promoting Arg-1 expression and repressing IL-16 and CXCL12 *in vitro* and *in vivo*. In addition, miR-210 overexpression or IL-16/CXCL12 silencing in MDSCs promoted breast cancer growth, whereas miR-210 inhibition reversed the T-cell suppression phenotype (168). In the case of miR-210, HIF-1α drives miRNA expression; however, miRNAs can reciprocally regulate HIF-1α expression. miR-155 degrades HIF-1α in MDSCs, inhibiting the immune suppressive effects. In murine melanoma and lung cancer, miR-155 KO mice exhibited decreased T-cell function and greater accumulation of MDSCs in the spleen and tumor. In addition, tumor and bone marrow miR-155 KO MDSCs are more proficient at migrating and increasing the production of chemokines that promote additional MDSC and PMN infiltration into the TME. The loss of miR-155 in MDSCs also enhanced suppression of T-cell function by upregulating Arg-1, iNOS, VEGF, and MMP2/MMP9 expression (169). Although miR-155 has a defined role in this study, its effects in MDSCs vary depending on the study and context. For instance, miR-155 promoted MDSC function and immune suppression in another study. Here, miR-155–competent animals exhibited enhanced T-cell infiltration, but the cytotoxic capacity of these T cells was suppressed by the increased frequency of MDSCs in tumors. The loss of miR-155 in MDSCs improved T-cell function and decreased *Arg-1*, *iNOS*, *Vegf*, and *Mmp-9* expression, all of which are involved in immune suppression, tumor vascularization, and metastasis. SOCS1 was upregulated in miR-155 deficient MDSCs and inhibited MDSC-mediated immune suppression (170). Seemingly contradictory findings regarding miR-155 function in MDSCs may be explained by different contexts and its relative functional importance in MDSCs versus other immune effector cell types, as multiple studies have shown that the net effect of miR-155 loss in mice results in increased tumor growth. Taken together, HIF-1α is induced by a TME-intrinsic property that regulates MDSCs through miRNAs; however, it is also important to examine the role of other critical MDSC regulatory factors.

TGF-β is another critical regulator of MSDC function. Although GM-CSF and IL-6 induce PMN-MDSCs and miR-155/miR-21, the addition of TGF-β greatly enhances the expression of these miRNAs. miR-155 and miR-21 repress SHIP-1 and PTEN, respectively, and promote MDSC suppression of T-cell function. In addition, downregulation of SHIP-1 and PTEN increases STAT3 phosphorylation, a critical TF for MDSC development (171). During PMN-MDSC development, Stat3 and Cebpβ induce miR-21a, miR-21b, and miR-181b to repress Wdr5, Ash2l, and Mll1, respectively (172). As such, Wdr5, Ash2l, and Mll1 are all simultaneously downregulated during PMN-MDSC development, as they inhibit PMN-MDSC proliferation, expression of Arg-1 and iNOS, and suppression of CD4+/CD8+ T cells and M1 macrophages. Wdr5, Ash2l, and Mll1 promote terminal differentiation of PMN-MDSCs into mature phagocytic proinflammatory neutrophils. Ultimately, the inhibition of miR-21a, miR-21b, or miR-181b or overexpression of Wdr5, Ash2l, and Mll1 in MDSCs promoted similar degrees of tumor control (172). TGF-β is also a potent inducer of miR-30a and miR-494 (173, 175). miR-30a expression is upregulated in MDSCs in mice with B-cell lymphoma (173). Overexpression of miR-30a led to increased levels of MDSCs, and inhibition of miR-30a reciprocally led to a loss of MDSCs. Functional validation experiments revealed that miR-30a degrades SOCS3, which inhibits MDSC development and function by promoting STAT3 phosphorylation (173, 174). miR-30a also promoted increased ROS, ARG-1, and IL-10, inhibiting the proliferation of and IFN-γ production from CD4+ T cells. In addition, specific agonism and antagonism of miR-30a in MDSCs led to increased and decreased tumor burden, respectively (173). miR-494 is highly expressed in TGF-β–induced MDSCs and led to increased expression of Arg-1, MMP2, MMP13, and MMP14 and degradation of PTEN (175). The loss of PTEN then led to increased activation of Akt, mTOR, and NF-κB, all of which promoted metastasis and proliferation. Thus, *in vivo* knockdown of miR-494 inhibited MDSC proliferation and function while restoring CD8+ T-cell activity, leading to increased survival, decreased tumor size, and metastatic burden (175). Ultimately, miRNAs dynamically change depending on the molecular signature of the TME and play developmental and functional roles in MDSCs.

To control MDSC development and function, miRNAs degrade TFs involved in nonpathological hematopoiesis. For example, miR-9 directly degrades Runx1, and miR-9 overexpression promoted MDSC function and inhibited anti-tumor immunity in a preclinical model of lung cancer. In human lung cancer, miR-9 and Arg-1 are positively correlated and associated with lung cancer tissue; whereas, Runx1 is associated with healthy tissue and negatively correlated with both miR-9 and Arg-1 (176). In addition, miR-34a increases the number and frequency of MDSCs in bone marrow and spleen by repressing N-myc, which promoted MDSC persistence by inhibiting apoptosis. Intriguingly, miR-34a overexpression promotes proinflammatory peripheral tissue MDSCs by downregulating the expression of Arg-1 and TGF-β and promoting the expression of TNF-a, iNOS, and IL-17, suggestive of an M1 phenotype. *In vivo* miR-34a overexpression slowed tumor growth, highlighting the importance of MDSC cytokine profiles (177). Taken together, miRNAs provide insight into the development and function of MDSCs and can potentially serve as therapeutic targets or agents.

## Immunomodulatory roles of tumor miRNAs

**Figure 7:**
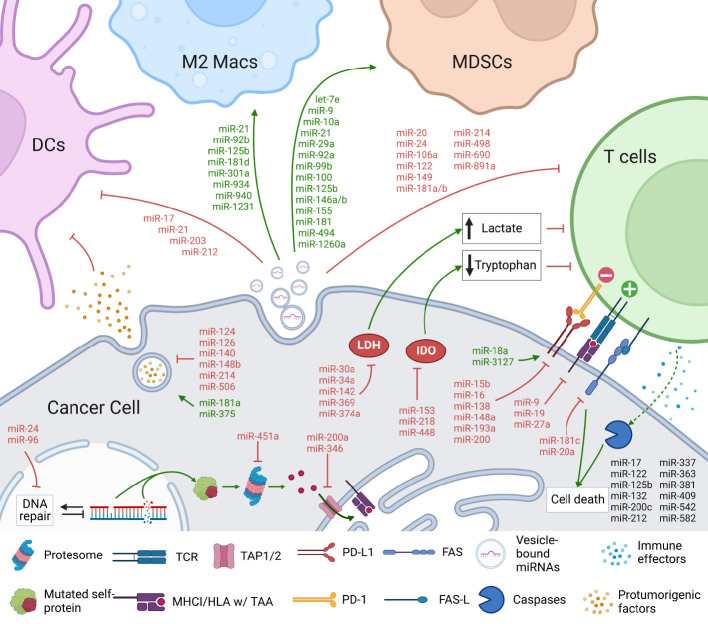
Tumor cell miRNAs regulate immune and cancer cell functions, influencing various aspects of anti-tumor immunity. Tumor-cell expressed miRNAs regulate various aspects of the anti-tumor immunity. Studies have shown that miRNAs can impact tumor antigenicity by controlling DNA repair and antigen presentation machinery. Furthermore, miRNAs can inhibit mediators of cell death, including caspases and death receptors, to prevent immunologic cell death. miRNA-mediated control of immunomodulatory cytokines and metabolic mediators such as lactate dehydrogenase (LDH) and indoleamine 2 3-dioxygenase (IDO) can alter the immune landscape within the tumor microenvironment. Importantly, tumor-derived miRNAs can be transported *via* extracellular vesicles into a variety of immune cells, including dendritic cells (DCs), macrophages (Macs), myeloid-derived suppressor cells (MDSCs), and T cells, and regulate their functions. Several miRNAs regulate multiple pathways simultaneously, suggesting that the critical miRNA regulatory hubs can be targeted to potentiate anti-tumor immunity.

miRNAs produced by tumor cells themselves regulate numerous immunoevasion mechanisms and are critical determinants in the immunological outcome in cancer (213). Mechanistically, tumor miRNAs regulate tumor responses that influence the immunological landscape in the TME, or are transferred through extracellular vesicles (EVs) to immune cells in the TME (214). Here, we will highlight key examples of immunomodulation by tumor cell–specific miRNAs and discuss their utility as clinical targets and disease biomarkers (**Figure 7** and **Table 3**).

**Table 3:** Tumor-derived and tumor miRNAs, targets, and functions.

***Tumor-derived* miRNAs that regulate dendritic cell (DC) function**			
***miRNA***	***Target***	***Function***	***Reference***
miR-17-5p	N/A	Reduces DC phagocytic potential, downregulates anti-tumorigenic IL-12, and upregulates of pro-tumorigenic IL-10 in gastric cancer	(215)
miR-21-5p	N/A	Inhibits DC activation and trafficking in esophageal and hepatocellular carcinoma	(216, 217)
miR-203	N/A	Inhibits TLR4 and downstream cytokine production (TNFa and IL-12)	(218)
miR-212-3p	RFXAP	Inhibits the transcription factor, RFXAP, necessary for MHCII expression	(219)
miR-155, miR-142, let-7i	SOCS1	Increases MHCII, CD80, and CD86 expression and DC maturation	(220)
***Tumor miRNAs* that indirectly regulate DC function**			
***miRNA***	***Target***	***Function***	***Reference***
miR-181a	SRCIN1	Promotes tumor stimulation of the SRC/VEGF signaling pathway in colorectal cancer	(221)
miR-140-5p	VEGF-A	Inhibits VEGF-A secretion, angiogenesis, and invasion of tumor cells in breast cancer	(222)
miR-126, miR-126*	N/A	Inhibits CCL2-mediate recruitment of inflammatory monocytes and mesenchymal stem cells, preventing breast cancer metastasis	(223)
***Tumor-derived* miRNAs that regulate macrophage function**			
***miRNA***	***Target***	***Function***	***Reference***
miR-301a-3p	N/A	Induces M2 macrophage polarization by activating the PTEN/PI3Kγ pathway and correlates to invasion, metastasis, and poor patient prognosis in pancreatic cancer	(224)
miR-21-3p, miR-125b-5p	SOCS4	Promotes M2 macrophage polarization by increasing p-STAT3 in epithelial ovarian cancer	(225)
miR-21-3p, miR-181d-5p	SOCS5		
miR-92b-3p, miR-1231-5p	PTEN	Activates and promotes phosphorylated AKT, STAT3, and STAT6 and subsequent M2 polarization in bladder cancer	(226)
miR-940	N/A	Promotes M2 macrophage polarization in epithelial ovarian cancer	(227)
miR-934	PTEN	Induces M2 macrophage polarization and premetastatic niche formation through CXCL13, activating a CXCL13/CXCR5/NFkB/p65/miR-934 positive feedback loop in colorectal cancer	(228)
miR-375	TNS3, PXN	Enhances macrophage migration and infiltration into tumor spheroids in breast cancer	(229)
***Tumor miRNAs* that regulate M2 TAM-mediated tumor progression**			
***miRNA***	***Target***	***Function***	***Reference***
miR-124	CCL2	Inhibits tumor promoting and metastatic potential in cancer associated fibroblasts and oral cancer cells	(230)
miR-506-3p	FoxQ1	Downregulates CCL2 production from colorectal cancer	(231)
miR-375	N/A	Induces CCL2 production from breast cancer	(229)
miR-214	CCL5	Reprograms normal fibroblasts into cancer associated fibroblasts in ovarian cancer	(232)
miR-148b	CSF1	Inhibits hepatocellular carcinoma growth and metastasis	(233)
***Tumor-derived* miRNAs that regulate MDSC function**			
***miRNA***	***Target***	***Function***	***Reference***
miR-146a/b, miR-155, miR-125b, miR-100, miR-99b, let-7e	N/A	Converts monocytes into MDSCs and promotes T-cell suppression and resistance against anti-CTLA and anti–PD-1 in melanoma	(234)
miR-10a	Rora		
miR-21	Pten	Promotes MDSC expansion and immune suppressive function in hypoxia-induced glioma	(235)
miR-9	SOCS3	Promotes development of early-stage-MDSCs, immune escape, and tumor growth through IL-6 in breast cancer	(236)
miR-181	PIAS3		
miR-29a, miR-92a, miR-155, miR-494, miR-1260a	N/A	Regulates MDSC function in cancer	(237)
***Tumor-derived* miRNAs that regulate T-cell function**			
***miRNA***	***Target***	***Function***	***Reference***
miR-24-3p, miR-891a, miR-106a-5, miR-20a-5p	N/A	Inhibits T-cell proliferation, differentiation, and anti-tumor cytokine production in nasopharyngeal carcinoma	(238)
miR-214	PTEN	Promotes Treg differentiation and function, promoting tumor growth in cancer	(239)
miR-3187-3p, miR-498, miR-122, miR149, miR-181a/b	N/A	Diminishes TCR signaling strength and TNFa production in melanoma	(240)
miR-690	Bcl-2	Activates mitochondrial apoptosis pathway in CD4+ T cells by activating caspase family proteins and downregulating BCL-2, MCL-1, and BCL-XL in melanoma	(241)
***Tumor-derived* miRNAs that regulate NK cells**			
***miRNA***	***Target***	***Function***	***Reference***
miR-23a	LAMP-1 (CD107a)	Inhibition of NK cell degranulation in hypoxic tumors	(242)
miR-296-3p	ICAM-1	Promotes metastasis by repressing NK cell ligands in prostate cancer	(243)
miR-153	HIF-1α, ADAM10	Inhibits shedding of MICA from the surface of cancer cells in pancreatic cancer	(244)
***Tumor miRNAs* that regulate antigen presentation**			
***miRNA***	***Target***	***Function***	***Reference***
miR-24	H2AX	Suppresses DNA repair	(245)
miR-96	REV1, RAD51		(246)
miR-451a	PSMB8	Inhibits prostate cancer cell proliferation, colony formation, and invasion and promotes apoptosis	(247)
miR-200a-5p	TAP1	Inhibits antigen processing and MHCI expression, leading to poorer patient outcomes in melanoma	(248)
miR-346	TAP1	Inhibits MHCI gene products, IFN-inducible genes, and TAP1	(249)
miR-19	N/A	Downregulates IFN-inducible genes and MHCI genes	(250)
miR-27a	Calreticulin	Inhibits MHC class 1 molecules and correlates with poor patient prognosis in colorectal cancer	(251)
***Tumor miRNAs* that regulate immune checkpoint ligands**			
***miRNA***	***Target***	***Function***	***Reference***
miR-148a-3p	PD-L1	Downregulates IFN-γ–inducible PD-L1 expression and immunosuppression in MSI-H colorectal cancer cell and anaplastic thyroid cancer	(252, 253)
miR-200	PD-L1	Inhibited by ZEB1 to enhance PD-L1 expression to promote epithelial to mesenchymal transition, metastasis, and T-cell dysfunction in lung cancer	(254)
miR-15b, miR-16, miR-193a-3p	PD-L1	Anticorrelates with and is predicted to target PD-L1. Low expression predicts poorer outcomes in malignant pleural mesothelioma	(255)
miR-138-5p	PD-L1, PD-1	Downregulates PD-L1 on cancer cells and DCs and PD-1 on T cells, inhibiting tumor cell immune evasion and enhancing anti-tumor immunity in non–small cell lung cancer	(256)
miR-18a	PTEN, WNK2, SOC6, BTG3, RBSP3	Promotes PD-L1–induced tumor proliferation, invasion, and tumorigenesis while inhibiting multiple tumor suppressor genes in cervical cancer	(257)
miR-3127-5p	N/A	Promotes STAT3 phosphorylation and subsequently upregulates PD-L1 in non–small cell lung cancer	(258)
***Tumor miRNAs* that regulate immunologic cell death**			
***miRNA***	***Target***	***Function***	***Reference***
miR-582-5p, miR-363	Caspase 3, Caspase 9, Bim	Promotes glioblastoma stem cell survival	(259)
miR-337-3p	Caspase 3	Decreases tumor necrosis factor-related apoptosis-inducing ligand (TRAIL)–mediated cytotoxicity in pancreatic cancer	(260)
miR-17-5p, miR-132-3p, miR-212-3p	Caspase 7		
miR-125b-5p, miR-200c-3p, miR-409-3p, miR-122-5p, miR-542-3p	Cyclins, Caspases, Bcl-2, Bcl-2 like genes	Promotes tumor cell apoptosis and cell cycle arrest in breast cancer	(261)
miR-381-3p	N/A	Inhibits activation of caspase 3/8 and apoptosis in renal cell carcinoma	(262)
miR-181c	FAS	Prevents cell growth, cell cycle, and apoptosis in Ewing’s sarcoma	(263)
miR-20a	N/A	Inhibits Fas by modulating the promoter region to promote tumor cell growth in osteosarcoma lung metastasis	(264)
***Tumor miRNAs* that regulate immunometabolites**			
***miRNA***	***Target***	***Function***	***Reference***
miR-153	IDO1	Improves CAR-T cell therapy in colon cancer	(265)
miR-218	IDO1	Inhibits cervical cancer cell viability and promotes apoptosis	(266)
miR-448	IDO1	Suppresses apoptosis of intratumoral CD8+ T cells, promoting improved anti-tumor immunity	(267)
miR‐34a/c, miR‐369‐3p, miR‐374a, miR‐30a‐5p, miR‐142‐3p	LDHA	Inhibits production of lactate and immune suppression	(268)
miR-342-3p	MCT1	Inhibits lactate and glucose flux changes in and decreases proliferation, viability, and migration of triple negative breast cancer	(269)
miR-124	MCT1	Increases intracellular pH and glycolytic activity to inhibit proliferation of pancreatic cancer	(270)

“N/A” is not available.

### Regulation of DC functions

DCs play key roles in educating and activating T cells against cancer cells and can be subverted within the TME, allowing cancer immunoevasion. Tumor miRNAs can, directly and indirectly, control DC maturation and induce a tolerogenic state. For instance, gastric cancer–derived miR-17-5p was taken up by DCs, leading to a reduction in phagocytic potential, downregulation of anti-tumorigenic IL-12, and upregulation of pro-tumorigenic IL-10 (215). Furthermore, miR-17 treatment of DCs promoted T-cell hyporesponsiveness and induced Tregs, suggesting a tolerogenic DC phenotype. miR-21-5p is another miRNA packaged into tumor EVs from esophageal and hepatocellular carcinoma cells (216, 217) and stroma (271), and upon DC uptake, inhibits DC activation markers and chemokine receptors required for proper trafficking. In pancreatic cancer, miR-203 and miR-212-3p are secreted in tumor EVs and inhibit DC activation by targeting TLR4 and RFXAP, a key TF required for MHCII induction, respectively (218, 219). Complementing these studies, another group showed improved DC maturation by electroporating tumor-derived exosomes with miR-155, miR-142, and let-7i, underscoring this mechanism’s importance in the induction anti-tumor immunity and its potential for therapeutic purposes (220).

In addition to DC miRNA uptake impacting their functions, miRNAs regulate immunomodulatory gene expression in tumor cells, indirectly inhibiting DC responses. Several tumor-secreted molecules inhibit DC functions in cancer, including VEGF, TGF-β, GCSF, CCL2, Wnt1a/5a/16b, and IL-6 (272), and oncogenic miRNA networks in tumor cells alter the expression of these genes. VEGF expression is induced by miR-181a in CRC cells (221) and reduced by miR-140 in breast cancer cells (222). In addition to negative regulatory roles in DCs, VEGF signaling within the TME can directly induce tumor growth, angiogenesis, and metastasis. Similarly, breast cancer metastasis can be promoted by increased recruitment of inflammatory cells in a CCL2-dependent manner, which was suppressed by miR-126/miR-126* in cancer cells (223). These studies suggest that oncogenic miRNA networks act through multiple mechanisms to reduce DC-mediated immunity and increase tumor fitness, and therapeutic targeting of key tumor miRNAs has strong therapeutic potential in the clinic.

### Regulation of TAMs

Macrophages can be defined by a continuum of activation states on the M1–M2 axis (273). Several groups have described the important roles of tumor-derived miRNAs in co-opting macrophage polarization mechanisms to enable tumor immunoevasion, progression, and metastasis. In hypoxia-treated pancreatic cancer cell exosomes, miR-301a-3p was enriched, which induced M2s through PI3K pathway activation (224). Moreover, circulating exosomal miR-301a-3p correlated with invasiveness and poor survival, suggesting a role for miR-301a-3p as a biomarker and a new clinical target. Similarly, hypoxic conditions enriched ovarian cancer cell exosomes for miR-21-3p, miR-125b-5p, and miR-181d-5p, which induced M2 polarization in culture through SOCS4/5/STAT3 pathways (225). AKT/STAT3-mediated M2 polarization was also induced by tumor-derived miR-92b-3p and miR-1231-5p in bladder cancer (226). Another study also implicated miR-940 in tumor-mediated induction of M2s in ovarian cancer (227). In metastatic CRC, tumor-expressed miR-934 led to increased production of CXCL13 in M2-like TAMs and activated a CXCL13/CXCR5/NFkB/p65/miR-934 positive feedback loop in cancer cells (228). It was suggested that this signaling cascade created a conducive pre-metastatic niche for the growth of CRC cells in the liver. Because the CXCL13/CXCR5 axis can orchestrate the immunological dynamics in the TME (274), it would be interesting to investigate the involvement of miR-934–mediated tumor-macrophage crosstalk in the systemic metastasis of other cancer types.

The enrichment of M2-like TAMs can also be mediated by secreted tumor factors under the direct regulation of miRNA networks. For instance, CCL2 induces monocyte infiltration into the tumors, which are subsequently subject to the pro-tumorigenic differentiation pathway (275, 276). The CCL2 transcript has evolutionarily conserved target sites for at least two miRNAs: miR-124 and miR-506. These miRNAs directly inhibited CCL2 expression in oral cancer (230) and colorectal carcinoma cells (231), promoting tumor suppression by reducing TAM recruitment and polarization. Conversely, miR-375 induced CCL2 expression in human breast cancer cell lines and was transferred to TAMs in apoptotic tumor bodies, directly regulating their polarization (229). This study described a robust positive correlation between miR-375 and CCL2 levels and miR-375 and TAM infiltration in invasive human breast cancer biopsies. Similarly, secretion of CCL5 from cancer-associated fibroblasts can induce TAM infiltration, which is critically regulated by miR-214 (232). CSF1 is another critical cytokine for the infiltration and maintenance of TAMs. Studies have shown that miR-148b serves as a tumor-suppressor by blocking CSF1 expression, subsequently inhibiting the TAM infiltration in hepatocellular carcinoma (233). These findings suggest that tumor miRNAs can regulate TAM phenotypes by functioning in tumor cells and macrophages upon intercellular transfer.

### Regulation of MDSC functions

MDSCs are a heterogeneous population of cells of myeloid origin that aid in tumor progression through various mechanisms, and tumor miRNAs can directly control MDSC recruitment and functions. A recent study described a set of miRNAs, including miR-146a/b, miR-155, miR-125b, miR-100, miR-99b, and let-7e, as inducers of MDSC-mediated immune suppression and ICB resistance in melanoma (234). These EV-derived miRNAs were transferred *via* melanoma EVs into CD14-expressing peripheral blood monocytes, leading to the acquisition of suppressive activity on T cells in culture. In complementary experiments, silencing these miRNAs in melanoma cells inhibited the induction of the MDSC immunosuppressive phenotype. Importantly, these MDSC-associated miRNAs were enriched in plasma samples of patients with melanoma who are refractory to ICB treatment, suggesting that they can be novel immunotherapy targets and potential biomarkers for stratifying patients. Other studies have shown that hypoxia-inducible miRNAs, including miR-10a and miR-21, can also be secreted in EVs that MDSCs take up in the TME to enhance the immunosuppressive features of these cells (235). The exosomal miR-10a and miR-21 repressed RORA and PTEN, leading to MDSC expansion and increased secretion of immunosuppressive molecules such as TGFb and IL-10. A recent study reported that miR-9 and miR-181a secreted in breast cancer exosomes inhibited SOCS3 and PIAS3, leading to JAK/STAT pathway activation in MDSCs (236). The study of exosomal miR-9 and miR-181a highlights a key mechanism of MDSC expansion and T-cell immunosuppression driven by IL-6 in breast cancer and targetable pathways to combat breast cancer-mediated immunosuppression (277). In addition to these miRNAs, cancer-derived miR-29a, miR-92a, miR-155, miR-494, and miR-1260a are involved in the regulation of MDSC functions in cancer (237). It is noteworthy that some of these miRNAs can regulate multiple cell types in the TME to facilitate immunoevasion and thus can serve as versatile therapeutic targets. In addition to the discussed miRNA networks, other miRNA networks regulating specific chemokines could potentially be therapeutic targets to inhibit MDSC recruitment to the TME (278).

### Regulation of T and NK cell responses

Because T cells and NK cells are key effector cells responsible for eliminating tumor cells, tumor miRNAs subvert several aspects of T- and NK-cell–mediated immunity. Nasopharyngeal cancer exosomes and their miRNA cargo, including miR-24-3p, miR-891a, miR-106a-5, and miR-20a-5p, regulated T-cell differentiation and proliferation (238). Importantly, tumor exosomes induced pro-tumorigenic Treg differentiation at the expense of the anti-tumorigenic Th1/Th17 differentiation, suggesting that tumor miRNAs can simultaneously mediate immunoevasion through multiple mechanisms. Another study involving multiple human and mouse cancers reinforced the roles of tumor-derived miRNAs in controlling Treg functions and suggested that microvesicle-bound miR-214 induced Treg differentiation by blocking PTEN expression (239). Furthermore, mesenchymal stem cells recruited to tumor sites aid in cancer progression and can secrete exosome-bound miRNAs to induce Treg differentiation, suggesting these multilayered regulatory mechanisms could be strong therapeutic targets (279). Certain tumor-derived miRNAs, including miR-3187-3p, miR-498, miR-122, miR149, and miR-181a/b, diminished T-cell receptor signaling strength and inhibited the production of TNFa, a key effector molecule in anti-tumor immunity (240). In addition to impacting T-cell differentiation and effector functions, tumor-derived miRNAs also directly regulate T-cell fitness and survival. For instance, tumor exosome-bound miR-690 activated the mitochondrial apoptosis pathway in helper T cells by downregulating BCL-2, MCL-1, and BCL-XL (241). Another study also suggested that melanoma exosomes impact mitochondrial respiration of CTLs, although the effects could have been mediated by their miRNA cargo, among other factors (280).

NK cells are essential for eliminating tumor cells that evaded CTL recognition. Unsurprisingly, cancer cells have evolved mechanisms to inhibit NK functions for sustained growth. Exosomes secreted by hypoxia-treated lung carcinoma and myeloid leukemia cells contained miRNAs, which interfered with NK-cell–mediated cytotoxicity (242). Specifically, miR-23a was enriched in hypoxic tumor exosomes, leading to the inhibition of the NK cell degranulation, evidenced by the reduced LAMP-1 (CD107a) expression. Another study showed membrane-bound TGF-β1 in tumor exosomes downregulating NK cell activation receptors and inhibiting NK cell function (281). In addition to regulating NK cell cytotoxicity, tumor miRNAs alter the expression of tumor ligands in a cell-intrinsic manner, controlling the negative and positive signals exerted on NK cells (282). ICAM-1 is among these ligands, which binds to LFA-1 on NK cells to deliver a positive signal. In prostate cancer, miR-296-3p directly targeted and degraded ICAM-1, resulting in cancer cells resistant to NK-mediated lysis (243). Hypoxia-induced immunoevasion of pancreatic cancer cells involved sponging of miR-153 by a circular RNA. Loss of miR-153 resulted in the shedding of MICA, a natural ligand for the NK surface activation molecule NKG2D, from the surface of cancer cell (244). Several other studies reported the role of tumor miRNAs in controlling the expression of other NKG2D ligands, including MHC class I polypeptide-related sequence B (MICB), UL16 binding protein (ULBP), and other immunological mediators (213, 282, 283). In this context, tumor miRNAs and their targets provide a roadmap to targetable pathways that may lead to success in the clinic.

### Regulation of tumor antigen presentation

T-cell–mediated cytotoxicity requires the presentation of peptide antigens on the tumor cells. A common mechanism of immunoevasion is the downregulation of tumor antigens and the cellular machinery associated with displaying the antigenic peptides. An optimal tumor antigen needs to be i) distinct from the self-endogenous peptides, ii) processed correctly and intracellularly, iii) loaded onto MHC molecules efficiently, and iv) have the structural properties to mediate a sufficiently long interaction between T cells and the antigen-presenting cell. Tumor miRNAs significantly impact the antigen presentation process at various stages. As the generation of tumor neoantigens is closely associated with genomic instability, miRNAs controlling DNA repair mechanisms can be directly involved in tumor antigenicity (284). For instance, miR-24 and miR-96 suppressed DNA repair by targeting H2AX and REV1/RAD51, respectively (245, 246). Conversely, other miRNAs function to maintain genome stability, reducing the antigenicity of the tumor (285). Inhibiting miRNAs that maintain genomic stability in tumor cells renders the tumor more immunogenic and susceptible to DNA-damaging chemotherapeutic agents. Proteasomes then degrade the mutant proteins into short peptides for antigen presentation in cancer cells. Proteasomes generate different repertoires of peptide products, depending on the composition of their subunits (286). Thus, miRNA regulation of proteasomes can determine tumor antigenicity. Deficiency of the immunoproteasome formed by PSMB7/8/9 subunits was associated with immune escape and poor outcomes in NSCLC (287). In prostate cancer, miR-451a inhibited the expression of PSMB8 (247), but the clinical significance of these miRNAs remains undetermined. Lastly, miRNAs impact antigen presentation by regulating the expression of the channel proteins required for transferring degraded cytosolic peptides into the endoplasmic reticulum for the subsequent loading onto MHCI molecules. Specifically, miR-200a-5p bound to the 3′-UTR of the transporter associated with antigen processing 1 (TAP1) transcript, leading to its suppression and poor clinical outcomes in patients with melanoma (248). Similarly, endoplasmic reticulum stress in cell lines induced miR-346, which inhibited TAP1 and subsequent antigen presentation (249). Alternatively, miRNAs can regulate the expression of MHC genes to reduce the tumor antigen presentation. Studies have reported that multiple miRNAs, including miR-9, miR-19, and miR-27a, can, directly and indirectly, inhibit MHC expression (250, 251, 288). Intriguingly, a study revealed that peptides loaded onto HLA class-I are primarily derived from transcripts containing miRNA-binding sites (289). These findings suggest that miRNAs play an essential role in tumor antigenicity by regulating various aspects of the antigen presentation process.

### Regulation of immune checkpoint ligands

Immune checkpoint signaling is a key mechanism in regulating T-cell responses. T-cell expressed checkpoint receptors provide activating or inhibitory signals, and their therapeutic targeting has yielded clinical success across multiple cancers (290). However, most patients do not benefit from ICB, underscoring the need to elucidate the compensatory signaling and alternative pathways. miRNAs can regulate the expression of immune checkpoint ligands and protect the tumors from T-cell–mediated lysis. PD-L1 is a primary ligand that engages with the negative checkpoint receptor PD-1 on T cells and is often elevated in malignant tumors (291). In various contexts, several miRNAs control PD-L1 expression (292). DNA mismatch repair–deficient colorectal carcinoma is associated with high immune activity and levels of IFN-γ, which induced PD-L1 transcription by downregulating miR-148a-3p (252). The miR-148a/PD-L1 axis also contributed to thyroid cancer immune evasion (253). miR-138-5p also inhibited PD-L1 expression in lung cancer cells and DCs in the TME, and miR-138-5p expression inhibited tumor growth (256). In another study, the miR-200/ZEB1 regulatory axis controls PD-L1 expression and metastasis of lung cancer cells in a Kras-driven oncogenesis model (254). Furthermore, increased PD-L1 expression was observed in mesothelioma tumors that lost miR-15b, miR-16, and miR-193a-3p expression, which correlated with worse survival (255). Oncogenic miRNAs can mediate the opposite effect to drive immunoevasion and induce PD-L1 expression in cancer cells. For instance, miR-18a induced PD-L1 expression in cervical cancer cells through activating PI3K, ERK, and WNT signaling pathways (257). Similarly, miR-3127-5p upregulated PD-L1 expression on lung cancer cells by promoting STAT3 phosphorylation, leading to chemoresistance (258). These studies underscore the importance of miRNAs in regulating immune checkpoint signaling in cancer and suggest that miRNA targeting can further enhance the therapeutic outcomes in checkpoint immunotherapy.

### Regulation of immunologic cell death

The killing of tumor cells by the immune system involves programmed cell death and phagocyte-mediated removal. miRNAs play essential roles in regulating cancer and immune cell death. Various apoptosis pathways converge in caspases, which are sequentially activated cysteine proteases that function as the executor of the cell (293). Several tumor miRNAs directly controlled the expression of caspases and other pro-apoptotic molecules, such as miR-582-5p and miR-363 in glioblastoma (259); miR-337-3p, miR-17-5p, miR-132-3p, and miR-212-3p in pancreatic cancer (260); miR-125b-5p, miR-200c-3p, miR-409-3p, miR-122-5p, and miR-542-3p in breast cancer (261); and miR-381-3p in renal carcinoma (262). Alternatively, miRNAs can target upstream mediators, such as the Fas death receptor, involved in the extrinsic apoptosis pathway. miR-181c and miR-20a are two examples of miRNAs regulating Fas receptor expression in sarcoma (263, 264). In addition to controlling T-cell–induced cell death, miRNAs regulate tumor-innate immune cell interactions. For instance, miR-27a can block the expression of calreticulin, which can be displayed on tumor cell surfaces as an “eat-me” signal (251, 294). These findings suggest that multiple facets of immunologic cell death are under the control of miRNAs, and their targeting could open new clinical research avenues.

### Regulation of immunometabolites

The metabolic landscape of the TME is a critical determinant in anti-tumor immunity. Tumor cells compete with the infiltrating immune cells for nutrients while secreting inhibitory metabolites that directly mediate immunoevasion (295). Tumor miRNAs regulate numerous anabolic and catabolic pathways to meet the bioenergetic demands of cell proliferation and establish a favorable metabolic state for their growth at the expense of the immune cells (296). The primary example of miRNA-mediated regulation of immunometabolites in cancer is indoleamine 2,3-dioxygenase (IDO), a class of enzymes responsible for metabolizing tryptophan, an essential amino acid, into kynurenine (297). Cancer cells can upregulate IDO and limit the amount of tryptophan in the TME, which, in turn, inhibits effector T cells and promotes Treg function. Thus, IDO is considered a critical metabolic switch in the anti-tumor immunity (297). miR-153, miR-448, and miR-218 directly inhibited IDO expression in colon and cervical cancer cells and served as tumor-suppressor miRNAs (265–267). These studies suggested that anti-tumor T-cell functions can be improved by expressing specific miRNAs in tumor cells, opening new possibilities for combination treatments involving miRNA mimics and adoptive T-cell transfer immunotherapy.

In addition to controlling amino acid catabolism, miRNAs regulate cancer cell utilization of glucose as the primary energy source. Glucose is taken up by the glucose transporters on the cell surface and subsequently broken down *via* oxidative phosphorylation or aerobic glycolysis (298). Specifically, aerobic glycolysis is favored in tumor cells, effector T cells, and proinflammatory M1s, making glucose metabolism a key regulator of immune function and tumor growth in the TME (299). miRNAs control glucose uptake, intracellular processing, and the secretion of intermediary metabolites, all of which play an important role in tumor cell metabolic fitness and in the anti-tumor immune response. The generation of lactate as a by-product of glycolysis is particularly important, as increased extracellular lactate inhibits T-cell function. High levels of lactate dehydrogenase (LDH), the enzyme responsible for generating lactate from pyruvate, is associated with poor patient outcome and immunosuppression in cancer (268). Several miRNAs target LDH, including miR‐34a/c, miR‐369‐3p, miR‐374a, miR‐30a‐5p, and miR‐142‐3p (268), and are implicated in anti-tumor immunity in various study contexts. Alternatively, miRNAs also impact the release of lactate into the TME by regulating monocarboxylate transporters (MCTs). miR-342-3p and miR-124 repressed MCT1 in triple negative breast cancer and pancreatic cancer, respectively, leading to altered metabolism and suppression of tumor growth (269, 270). Targeting tumor glycolysis also improved anti–PD-1 therapy in melanoma by increasing the production of IFN-γ in T and NK cells (300). Taken together, miRNA regulation of tumor metabolic fitness impacts immune cell function within the TME. Thus, targeting metabolic regulators and/or their associated miRNAs may provide powerful approaches to eliminating tumors. However, one key challenge is targeting tumor metabolism while sparing the immune cells that utilize similar metabolites.

## miRNAs and cancer immunotherapy

Although immunotherapy has positively changed the outlook in advanced cancers, not all patients benefit from immunotherapy due to incompletely understood mechanisms. As described above, miRNAs play key roles in tumor immunity through their functions in both tumor and immune cells, highlighting their potential in disease classification and as therapeutic agents (301). Various classes of immunotherapy were shown to impact the miRNA networks in the TME (302–304). In this section, we will highlight the involvement of miRNAs in immunotherapy action mechanisms and discuss opportunities to improve clinical outcomes.

### Immune checkpoint blockade

Since 2015, ICB has gone from being a last-resort treatment to first-line therapy for specific patients. α-PD-1 monoclonal antibodies alone are used to treat over 18 cancer types and are indicated for three biomarker-based indications, including tumors with high microsatellite instability (MSI-H), deficient mismatch repair (dMMR), or high tumor mutation burden (TMB-H) (127, 128). FDA-approved ICB therapies, PD-1, PD-L1, CTLA-4, and LAG-3, have been used in combination with chemotherapy, α-VEGFA monoclonal antibody, mitogen-activated protein kinase kinase (MEK) inhibitor, B-rapidly accelerated fibrosarcoma (BRAF) inhibitor, hedgehog pathway inhibitor, and other ICB agents. Despite the tremendous progress over the last decade, ICB fails in most patients that fall outside the specified clinical classifications and show mediocre results depending on the cancer type.

Combination therapy has extended the survival of advanced-stage patients with the poorest outcomes, including treatment-refractory hepatocellular carcinoma, extensive-stage small cell lung cancer, nonresectable mesothelioma, among many others (305–307). The triumphs of combination therapy may be attributed to targeting multiple distinct pathways or priming an inflammatory response by tumor damage-associated molecular patterns (308). miRNAs can also target numerous pathways similarly. Biochemical validation has demonstrated the capacity of a single miRNA to target several hundred genes, depending on the cell type and context (54). As previously stated, miR-138 inhibits PD-1 and CTLA-4 expression (85), whereas mir-28 decreases PD-1, TIM-3, and BTLA expression (87). Unlike miR-138 and miR-28, miR-155 represses CTLA-4 and PD-L1 expression (54, 88). Reciprocally, miR-146a, an anti-inflammatory miRNA, promotes PD-1, CTLA-4, TIM-3, and LAG3 expression (86). In addition to checkpoint molecule regulation, miRNAs such as miR-155 promote T-cell–mediated tumor immunity through overlapping pathways with ICB (46). Thus, modifying T cells with miRNAs provides a potential method for targeting multiple checkpoint molecules without the adverse events related to using one and two ICB agents.

### Autologous T-cell transfer and CAR-T cells

T cells play an undeniable role in tumor rejection; however, the field continues to face challenges in harnessing the full potential of transplanting autologous anti-tumorigenic T cells. Originally, autologous T cells were isolated from melanoma resections, expanded *in vitro* with IL-2, before transplantation into patients with advance-stage melanoma. In the initial trials, nine out of 15 patients showed an objective response and regression of cancer in multiple organs (309). Since then, autologous T-cell transfers have undergone several variations to optimize tumor specificity and *in vivo* persistence leading to the development of chimeric antigen receptors (CARs). CARs are fusion proteins consisting of an antibody-based extracellular target recognition domain and intracellular costimulatory signaling domains (such as CD3, CD28, and 4-1BB) to generate tumor-antigen reactive T-cell clones. Defining the most effective CAR composition is an active area of research, and the most recent CAR–T-cell therapeutics have attained unprecedented clinical success (310–313). However, striking initial results are often followed by tumor recurrence after CAR–T-cell therapy due to tumor cell–intrinsic and tumor cell–extrinsic mechanisms. Current limitations of CAR–T-cell therapy include antigen escape, target antigen expression on non-tumor cells, trafficking and infiltration into tumors, immune suppression in TMEs, and CAR–T-cell–associated toxicities (314).

miRNAs may be a tool to address the challenges that CAR–T-cells face, because miRNAs can control tumor antigenicity, amplify T-cell responses, expand CTLs and anti-tumorigenic CD4+ T cells, increase T-cell infiltration into the TME, and counteract the effects of immunosuppression. For instance, miR-155 induced stem-like qualities in tumor-specific CD8+ T cells by limiting terminal differentiation, which enhanced T cell–mediated immunity and limited exhaustion/senescence (315). Thus, limiting terminal differentiation through miR-155 may abrogate CAR–T-cell irAEs while augmenting tumor immunity, as miR-155 enhanced many mechanisms of anti-tumor CTL and Th1 functions. In this review, we discuss many other miRNAs that can impact T-cell function within the TME and may be potential targets or therapeutic agents to optimize CAR–T-cell function in patients. Importantly, miRNA manipulation in CAR–T cells has significant and practical clinical potential, as the generation of CAR–T cells requires *ex vivo* engineering of a patient’s autologous T cells.

### Myeloid targeting therapeutics

Myeloid cells are a heterogeneous population of immune cells found in the TME, including monocytes, TAMs, MDSCs, and neutrophils. Myeloid cells can be found in various states and often carry out pro-tumorigenic functions within the tumor immune microenvironment. Because of their roles in modulating immune responses and their impact on tumor growth, myeloid cell subsets are attractive immunotherapy targets. In particular, targeting TAMs by inhibiting the colony-stimulating factor 1 receptor (CSF1R) axis has gained significant traction, and multiple clinical trials are underway involving drugs that block CSF1R and its ligands (316). Because TAMs are highly heterogeneous depending on the cancer type (317), whether CSF1R inhibition can benefit a broad range of patients with cancer remains unknown. Notably, miR-21 and 29a contain binding motifs for ETS, a downstream TF induced by CSF1R signaling, and miR-21 and miR29a expression is dependent on ETS. Functionally, miR-21 and miR-29 enhance tumor growth, inhibit M1s, promote M2s, and positively correlate to metastatic burden and poorer patient outcomes (165). Because miR-21 and miR-29 specifically enhance protumorigenic macrophages downstream of the CSF1R pathway, these miRNAs may be therapeutic targets with reduced off-target effect.

As described above, miRNAs are key regulators of macrophage functions. Thus, miRNA therapeutics have considerable clinical potential to synergize with TAM-targeting therapies and render the TME uniquely susceptible to such therapies (303). For instance, let-7b is upregulated in TAMs in prostate cancer, which correlated with an immunosuppressive M2-like phenotype (318). Studies have suggested the let-7 family as potential clinical targets, but their targeting needs to be done in a cell-specific manner because of their pleiotropic functions (319). Let-7 family may also be involved in regulating MDSC functions in cancer (320), and their targeting could further improve outcomes of MDSC-specific therapeutics, including CCR5 and CXCR2 blockers (321). The combination of miRNA therapeutics with approved or experimental immunotherapies is essentially an unexplored area with great promise for improving clinical outcomes in cancer.

### Oncolytic viruses

Oncolytic viruses (OVs) are a new class of immunotherapy that aims to eliminate tumor cells with infectious viruses and induce subsequent immune responses against released tumor antigens. The molecular characteristics of cancer cells, including downregulation of the IFN signaling pathway and other antiviral mechanisms, render them selectively susceptible for viral propagation (322). Although various classes of viruses have been proposed as OVs, adenoviruses are the most common infectious agent in clinical trials (323). The objective responses for OV therapy remain low (~9%). However, the disease was controlled in ~21% of the trial participants (323), suggesting significant room for improvement in OV therapy. miRNAs are critical regulators of the antiviral response, and selective targeting of cancer cells can potentially increase the OV efficacy. To that end, miRNA inhibitors or mimics can be delivered into the TME using intratumoral injections or liposome-mediated transfer. Alternatively, specific miRNA recognition sequences can be inserted into 3′-UTRs of OV genes to block their translation in healthy cells while allowing their expression in tumor cells (324). These approaches can expand the therapeutic window by increasing selectivity and efficacy simultaneously. The ongoing challenge in this context is identifying the right miRNA networks to leverage in OVs, which can only be achieved by better understanding the antiviral immune response. Although the high throughput gene expression profiling has been essential for characterizing the molecular landscape during viral infections, we must ultimately distinguish the miRNAs induced as part of the host response to the virus from the miRNAs induced by viruses themselves to enable their propagation. Such insight will be obtained through mechanistic studies involving sophisticated research models and the integration of multiple disciplines, including immunology, infectious disease biology, and bioinformatics.

### Therapeutic cancer vaccines

The potential of vaccines in inducing a robust immune response and our increased knowledge about the anti-tumor mechanisms have placed cancer vaccines under the spotlight as a new therapeutic approach. The aim of therapeutic cancer vaccines is to educate the immune system against specific TAAs in the presence of immune adjuvants to overcome the immunosuppression within the TME and eliminate cancer cells (325). TAAs, such as DNA, RNA, or peptides, can be directly injected into the patients or loaded onto APCs *ex vivo*, which are then reinfused into patients to stimulate anti-tumor immune responses (325). Although many types of vaccines are in development, there is currently only one approved therapeutic vaccine against prostate cancer known as sipuleucel-T (326). Sipuleucel-T involves *ex vivo* pulsing of peripheral blood DCs with a prostate-specific protein [prostatic acid phosphatase (PAP)] and GM-CSF before reinfusing these processed DCs in to the patient. Although the survival benefit provided by sipulecel-T was sufficient to obtain FDA clearance, the 36-month survival probability of the treatment group was 31.7% compared with 23% in the placebo group (326). Moreover, systemic cytokine-mediated adverse events were detected in most patients and the time to disease progression was similar between the two groups, suggesting a durable response is not attainable with the current approach. The treatment failure can be explained by tumor-intrinsic and tumor-extrinsic factors, including tumor antigen loss, inefficient antigen processing, suboptimal peptide binding to specific HLA haplotypes, barriers preventing T-cell infiltration into the tumor, and other immunosuppressive effects within the TME (327). In this context, blocking immunosuppressive miRNA networks in macrophages, MDSCs, and DCs can enhance immune responses to cancer vaccines. Upregulating miRNAs that positively regulate the immune response can also increase tumor clearance and overcome immunosuppression. This has been previously demonstrated *in vivo* with a DC-based vaccine containing a miR148a inhibitor, poly I:C (TLR3 agonist), and tumor antigen, which promoted enhanced anti-tumor immunity and survival by expanding mature DCs and suppressing Treg and MDSC development (150). *Ex vivo* handling of APCs or direct injection of genetic material into patients offers unique advantages in the delivery of miRNA inhibitors or mimics. Although the miRNA networks that can be safely modulated as vaccine adjuvants remain undetermined, we believe this area presents unique opportunities for combining immunotherapy with miRNA therapeutics to combat cancer.

## Concluding remarks

Despite the advances in immunotherapy, the field still faces challenges in the classification of patient populations, treatment-refractory cancers, low response rates, and irAEs, among many other criteria. However, miRNA-regulated biology provides an avenue to explore and understand tumor and immune cell interactions, many of which are targeted by current clinically used immunotherapies. This review highlights the role of immune and cancer cell–derived miRNAs, and the ways miRNAs can be leveraged to improve immunotherapy outcomes, as miRNAs in specific cell types provide resolution into pro-and anti-tumorigenic factors that influence patient outcomes. Here, we describe immunomodulatory miRNA networks and their targets regulating immune and cancer cell dynamics. Insights from the described studies may be utilized in patients with cancer to create more defined subsets with optimized treatment regimens. In addition, many miRNAs and their targets have been the focus of many preclinical studies to improve cancer outcomes. For example, miR-155 is broadly associated with a better anti-tumor response across all cancer types and has been leveraged in preclinical models in an immune cell–specific manner to improve anti-cancer immune responses. Meanwhile, many cancer cell–expressed miRNAs cripple anti-tumor immune responses, highlighting the multifaceted functions of miRNAs in different cell types. Thus, several lines of research focus on delivery systems, including extracellular vesicles and nanoparticles, that can direct miRNA mimics and/or inhibitors toward specific cells in the TME. Such selective targeting of miRNAs will undoubtedly help miRNA therapeutics reach their full clinical potential. However, miRNA therapeutics can still find applications without highly selective delivery methods because immune and tumor cells may have different functional thresholds for the targeted miRNA. Specifically, there may be therapeutic windows for non-selective miRNA manipulation to improve anti-tumor immunity and long-term clinical outcomes. Mechanistic studies of miRNAs in clinically relevant research models are indispensable for defining miRNA-dependent disease characteristics and targetable molecular mechanisms. Currently, miRNAs are underutilized in clinical practice as potential biomarkers and therapies. The continued pursuit of translational miRNA-based therapy will allow us to harness the potential of miRNAs to combat cancer and other disease states in the clinic. Looking toward the future, miRNA-controlled mechanisms of anti-tumor immunity will continue to shape therapeutic approaches in cancer.

## Author contributions

WT, HE, and RO: conceptualization, investigation, literature compilation, analysis, writing, reviewing, and editing. KB: conceptualization, reviewing, and editing. CB: conceptualization, reviewing, and editing. All authors contributed to the article and approved the submitted version.

## Funding

This work is supported by the Nation Institutes of Health 1F30CA260977 (WT), T32 Al138945 (KB), and 1F31CA261096 (CB).

## Acknowledgments

The authors acknowledge and thank Melanie Hall from the University of Utah writing center for assistance with editing this manuscript. All figures were made with the aid of Biorender.com.

## Conflict of interest

The authors declare that the research was conducted in the absence of any commercial or financial relationships that could be construed as a potential conflict of interest.

## Publisher’s note

All claims expressed in this article are solely those of the authors and do not necessarily represent those of their affiliated organizations, or those of the publisher, the editors and the reviewers. Any product that may be evaluated in this article, or claim that may be made by its manufacturer, is not guaranteed or endorsed by the publisher.

## Glossary

**Table:**

TME	tumor microenvironment
NK cells	natural killer cells
DCs	dendritic cells
RISC	RNA-induced silencing complex
TCR	T-cell receptor
MHC	major histocompatibility complex
mAb	monoclonal antibody
CTL	cytotoxic T lymphocyte
PFN	perforin
GZMB	granzyme B
CAR	chimeric antigen receptor
B-ALL	B-cell acute lymphoblastic leukemia
KO	knockout
TF	transcription factor
SOCS1	suppressor of cytokine signaling-1
PD-L1	programmed death ligand-1
PD-1	programmed cell death protein-1
Th1	T helper cell type 1
Th2	T helper cell type 2
Treg	T regulatory cell
AC9	adenylyl cyclase 9
Th17	T helper cell type 17
ETBF	enterotoxigenic Bacteriodes fragilis
EAE	experimental autoimmune encephalitis
IL-23R	IL-23 receptor
ICB	immune checkpoint blockade
KIR	killer gene Ig-like receptors
DAP12	DNAX-activating protein 12
aKIR	allelic variants of KIR
PRF1	perforin-1
APC	antigen-presenting cell
MHCI	major histocompatibility complex class I
MHCII	major histocompatibility complex class II
TLR	Toll-like receptors
Arg2	Arginase-2
CD40L	CD40 ligand
IFNs	interferons
CaMKII	calcium/ calmodulin-dependent protein kinase II
TADCs	tumor-associated DCs
DNMT1	DNA methyltransferase 1
MDSC	myeloid-derived suppressor cell
TAA	tumor-associated antigen
IMDCs	immature myeloid dendritic cells
cDC1s	type 1 conventional dendritic cells
FLT3L	FLT3 ligand
NSCLC	non–small cell lung cancer
CCL2	C-C motif chemokine ligand 2
CSF1	colony-stimulating factor 1
TAMs	tumor-associated macrophages
iNOS or NOS2	inducible nitric oxide synthase
HSCs	hematopoietic stem cells
IFN-g	interferon-g
IFN-gR	interferon-g receptor
PPRE	PPARy regulatory elements
PMN-MDSCs	polymorphonuclear myeloid-derived suppressor cells
M-MDSCs	monocytic myeloid-derived suppressor cells
LOX1	lectin-type oxidized LDL receptor 1
EVs	extracellular vesicles
IDO	indoleamine 2,3-dioxygenase
LDH	lactate dehydrogenase
MCTs	monocarboxylate transporters
MSI-H	high microsatellite instability
dMMR	deficient mismatch repair
TMB-H	high tumor mutation burden
CSF1R	colony-stimulating factor 1 receptor
OV	oncolytic virus
PAP	prostatic acid phosphatase
